# Microenvironment Self‐Adaptive Nanoarmor to Address Adhesion‐ and Colonization‐Related Obstacles in Impaired Intestine Promote Bacteriotherapy Against Parkinson's Disease

**DOI:** 10.1002/advs.202510628

**Published:** 2026-01-14

**Authors:** Limeng Zhu, Yuyu Wu, Yingjie Chen, Xiyi Chen, Xingjie Zan, Yanlong Liu, Wujun Geng

**Affiliations:** ^1^ Wenzhou Key Laboratory of Perioperative Medicine Wenzhou Institute University of Chinese Academy of Sciences Wenzhou Zhejiang P. R. China; ^2^ School of Mental Health Wenzhou Medical University Wenzhou Zhejiang P. R. China; ^3^ School of Medicine Zhejiang University Hangzhou Zhejiang P. R. China; ^4^ Cixi Biomedical Research Institute Wenzhou Medical University Wenzhou Zhejiang P. R. China; ^5^ Department of Anesthesiology Wenzhou Central Hospital Affiliated to Wenzhou Medical University Wenzhou Zhejiang P. R. China; ^6^ Department of Pain The First Affiliated Hospital of Wenzhou Medical University Wenzhou Zhejiang P. R. China; ^7^ Oujiang Laboratory (Zhejiang Lab for Regenerative Medicine, Vision and Brain Health) Wenzhou Medical University Wenzhou Zhejiang P. R. China

**Keywords:** bacterial colonization, bacterial therapeutics, chitin‐like fragment‐CHI3L1 interaction, microenvironment self‐adaptive, Parkinson's disease

## Abstract

Intestinal localized inflammations are recognized as key contributors to the incidence and progression of diverse extraintestinal disorders. Probiotic colonization has been increasingly highlighted for its potential to modulate susceptibility and progression of such diseases. Considering the adhesion‐ and colonization‐related challenges posed by multiple physiological and pathological characteristics in the intestine, a microenvironment self‐adaptive nanoarmor is developed. Partially acetylated chitosan oligosaccharides (CS) were employed to tune the adaptability and responsiveness of nanoarmor, enabling efficient interaction with the intestinal interface under dynamic conditions. Notably, Chitinase‐3‐like protein 1 (CHI3L1), an inflammation‐related secreted glycoprotein, served as a colonized niche to facilitate probiotics colonization in pathological microenvironments by leveraging the specific interaction between chitin‐like fragment and CHI3L1. By combining the intestinal microenvironment self‐adaptive nanoarmor with the inherent anti‐inflammatory properties of *Lactobacillus plantarum* ST‐III (*L. plantarum*), the nanocoated bacteria demonstrated significantly improved performance in alleviating intestinal mucosal inflammation, restoring gut barrier integrity, and reestablishing microbial homeostasis. Furthermore, the nanocoated bacteria showed significant therapeutic potential in treating Parkinson's disease (PD), a model for extraintestinal disorders, as evidenced by their ability to improve motor behavior disorders, reduce dopaminergic neuronal death, and mitigate neuroimmune responses. This approach proposes new insights into the living therapeutics for the treatment of extraintestinal diseases.

## Graphical abstract

A microenvironment self‐adaptive nanoarmor is developed to effectively address the adhesion‐ and colonization‐related challenges posed by multiple physiological and pathological characteristics in the intestine. *L. plantarum*@MPN@CS showed significant therapeutic potential in treating Parkinson's disease (PD), a model for extraintestinal disorders, as evidenced by their ability to improve motor behavior disorders, reduce dopaminergic neuronal death, and mitigate neuroimmune responses.

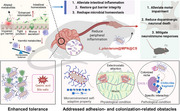

## 1 Introduction

The gut microbiota, a complex community of microorganisms, plays a critical role in maintaining intestinal homeostasis [1]. Beyond its essential role in gut health, it serves as a key determinant in the pathophysiological alterations of various extraintestinal organs, including the brain, liver, heart, lungs, kidneys, bone, and endocrine systems [2]. Intestinal inflammation has emerged as a pivotal contributor to the incidence and progression of diverse extraintestinal disorders, like Parkinson's disease (PD), fatty liver disease, chronic kidney disease, and diabetic nephropathy [3]. Substantial evidence from both clinical and preclinical studies highlights the presence of altered intestinal permeability and inflammation in these conditions [4, 5, 6]. The persistent translocation of toxins and pathogens from the gut into the systemic circulation leads to tissue damage in distant organs, thereby perpetuating a vicious cycle that exacerbates these disorders [7]. Given this intricate interplay, strategies that targeting the intestinal microbiota to effectively attenuate intestinal inflammation and restore gut barrier integrity hold significant potential for modulating extraintestinal disorders.

Oral delivery of probiotics has garnered increasing interest as an effective biotherapeutic strategy for maintaining gut homeostasis and managing related diseases, owing to its high patient compliance [8]. Growing evidence highlights the critical role of probiotic colonization in the gut mucus layer in modulating disease susceptibility and progression [9]. Despite significant advancements in formulation development, orally delivered bacterial‐based therapies still face substantial challenges that hinder their clinical application. The primary issue lies in the limited resistance of probiotics to the harsh conditions within the gastrointestinal tract (GIT), such as acidic environments, bile acids, and enzymatic degradation, which significantly reduce their bioavailability [10]. Additionally, the physiological characteristics of the GIT, including rapid peristalsis, result in low microbial colonization efficiency, leading to inadequate therapeutic responses and prolonged treatment durations [11]. These challenges are further exacerbated by pathological conditions associated with diseases, which increase barriers to probiotic adhesion and colonization [12]. Key pathological features include exhaustion of mucus characterized by increased permeability, elevated localized oxidative stress with heightened levels of reactive oxygen species (ROS), and competition for ecological niches with disordered gut microbiota [13, 14]. These factors collectively hinder the effective probiotic colonization in the GIT, diminishing their therapeutic efficacy. Therefore, improving the ability of probiotics to resist environmental stressors and adhere to the intestine under both physiological and pathological conditions is crucial to enhancing therapeutic outcomes.

Engineered probiotic delivery systems have emerged as a promising approach to address these challenges and enhance the therapeutic efficiency of oral probiotic delivery [15, 16]. These systems utilize diverse interactions, including hydrogen bonding, electrostatic forces, hydrophobic interactions, and *π–π* conjugation, to improve in vivo retention at target sites [17]. However, existing engineered systems primarily focus on shielding probiotics from gastrointestinal stressors, often overlooking the substantial colonization barriers imposed by disease‐associated pathological conditions. Consequently, ingested probiotics often show limited ability to effectively colonize inflamed sites and are rapidly cleared from the gastrointestinal tract, leading to reduced bioavailability and diminished therapeutic efficacy. Given these limitations, there is an urgent need for strategies that not only address gastrointestinal challenges but also enhance probiotic colonization and persistence in pathological environments. Such advancements could lead to more effective probiotic‐based therapies, particularly for extraintestinal disorders, by optimizing the crosstalk between the gut microbiome and distant organs.

Microbiota‐host interactions are central to regulating bacterial colonization behaviors [18]. Intestinal microbial colonization is largely dictated by the interactions between bacterial surfaces and the intestinal biological interface [19, 20]. The structural and functional properties of bacterial surfaces directly influence key physiological processes, including adhesion, proliferation, and differentiation [21]. Consequently, strategies that modulate interfacial interactions between bacteria and their surroundings are crucial for controlling microbial colonization. A comprehensive review of the existing literature reveals that previous research has predominantly focused on enhancing bacterial interactions with surrounding interfaces, particularly the mucus layer and epithelial cells, which serve as colonized niches [22, 23]. However, depletion of the mucus layer significantly impairs these interactions, hindering probiotic adhesion and subsequent colonization [24]. Moreover, an increase in epithelial‐associated microbial communities has been shown to disrupt the intestinal barrier, exacerbating immune responses [25]. Given these challenges, there is an urgent need to identify and target alternative niches that can interact with probiotics, facilitating their ability to overcome competitive inhibition and enhance colonization in conditions of localized intestinal inflammation. Chitinase‐3‐like protein 1 (CHI3L1), also known as YKL‐40 in humans and BMP‐39 in mice, is a glycoprotein from the glycoside hydrolase family [26], which is upregulated during various inflammatory conditions [27]. Despite its classification within this family, CHI3L1 lacks enzymatic activity and instead binds chitin via a carbohydrate‐binding motif, facilitating specific interactions with chitin‐related molecules [28]. We hypothesize that overexpression of CHI3L1 could provide a favorable niche for targeted bacterial colonization in pathological environments. In this study, we try to employ cationic, partially acetylated chitosan oligosaccharides (CS) containing chitin‐like fragments to enhance the adaptability and responsiveness of nanoarmors, facilitating efficient interaction with the intestinal interface in dynamic microenvironments.

**SCHEME 1:**
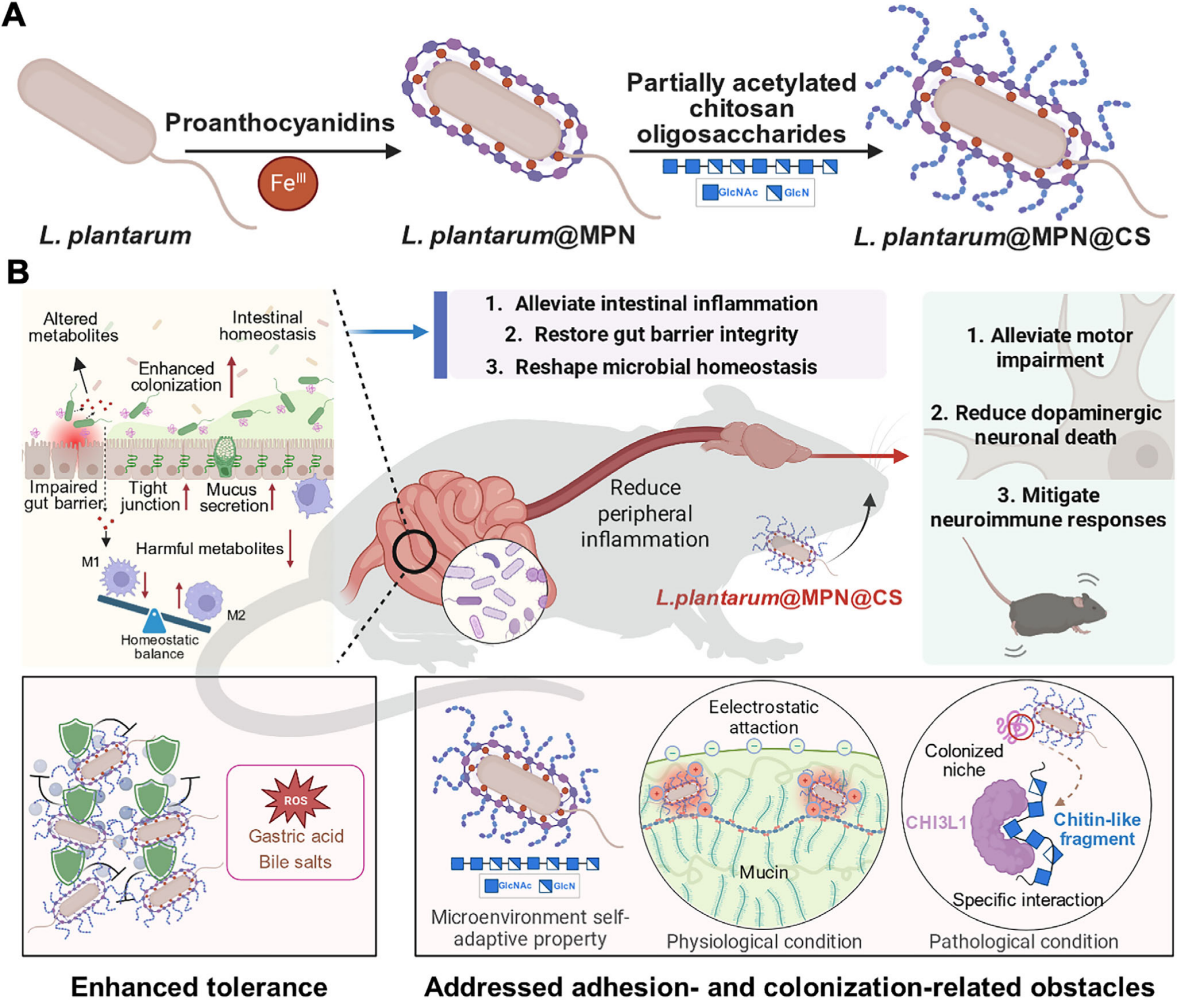
This schematic illustrates the key stages in functionalizing probiotics with a microenvironment self‐adaptive nanoarmor and explains the mechanisms by which the nanocoating enhances tolerance and promotes improved colonization for Parkinson's disease (PD) biotherapy. (A) The synthesis of *L. plantarum*@MPN@CS is shown, employing a layer‐by‐layer coating technique to construct the nanostructure. (B) The microenvironment self‐adaptive nanoarmor enhances probiotic viability and supports robust intestinal colonization in complex and dynamic physiological and pathological environments. It serves as an effective platform for modulating host‐microbiota interactions, offering a promising approach for advancing targeted therapies for extraintestinal conditions, as exemplified by its application in PD management. The figure was created with BioRender.com.

Here, we introduce an intestinal microenvironment self‐adaptive nanoarmor designed to regulate bacterial colonization and demonstrate its potential in developing bacterial therapeutics. Specifically, this nanoarmor is engineered to target gut microbiota and effectively alleviate intestinal dysbiosis, with the ability to alleviate extraintestinal disorders, such as PD. To address challenges related to complex operations, low yield rates, and mechanical damage to probiotics, we employed a simple, efficient, and biocompatible metal‐phenolic network (MPN) for encapsulating probiotics. The MPN nanocoating not only shields the probiotics from external stressors but also improves their adhesion within the intestinal environment. To further optimize interactions with the intestinal microenvironment, we incorporated cationic, partially acetylated CS, enabling surface charge reversal. This modification imparts a positive charge to the nanoarmor, facilitating robust electrostatic interactions with the negatively charged mucus layer under physiological conditions. Moreover, the inclusion of partially acetylated CS addresses adhesion and colonization challenges in pathological microenvironments by leveraging the specific recognition of chitin‐like fragments by CHI3L1. As a proof‐of‐concept study, we selected *Lactobacillus plantarum* ST‐III (*L. plantarum*) as the representative probiotic strain due to its well‐documented anti‐inflammatory, antioxidant, and endoplasmic reticulum stress‐relieving properties, positioning it as a highly promising therapeutic candidate for intestinal disorders [29]. The combination of the self‐adaptive nanoarmor with the inherent anti‐inflammatory properties of *L. plantarum* enabled the nanocoated bacteria to alleviate intestinal inflammation, restore gut barrier integrity, and reestablish microbial homeostasis. Additionally, the nanocoated bacteria demonstrated significant therapeutic potential in treating PD, as evidenced by improvements in motor behavior, reductions in dopaminergic neuronal death, and mitigation of neuroimmune responses in a mouse model. We anticipate that this nanocoated bacterial platform, equipped with its microenvironment self‐adaptive nanoarmor, offers a novel strategy to address adhesion and colonization challenges in pathological environments. This strategy not only facilitates more effective regulation of gut‐distal organ crosstalk but also provides new insights into the treatment of extraintestinal diseases (Scheme 1).

## 2 Results and Discussion

### 2.1 Preparation and Characterization of *L. plantarum*@MPN@CS

*Lactobacillus* species, which is a widely recognized probiotic that naturally resides in the healthy gastrointestinal tract, plays a crucial role in maintaining the homeostasis of the intestinal microbiota [30]. Beyond their established contributions to gut health, certain *Lactobacillus* strains have shown promising therapeutic potential in mitigating neurological inflammation via gut‐brain axis modulation [31, 32]. In this study, *Lactobacillus plantarum* ST‐III (*L. plantarum*) was chosen for its established anti‐inflammatory, antioxidant, and endoplasmic reticulum stress‐relieving properties, positioning it as a promising candidate for modulating extraintestinal disorders by attenuating intestinal inflammation and restoring intestinal homeostasis [29].

**FIGURE 1:**
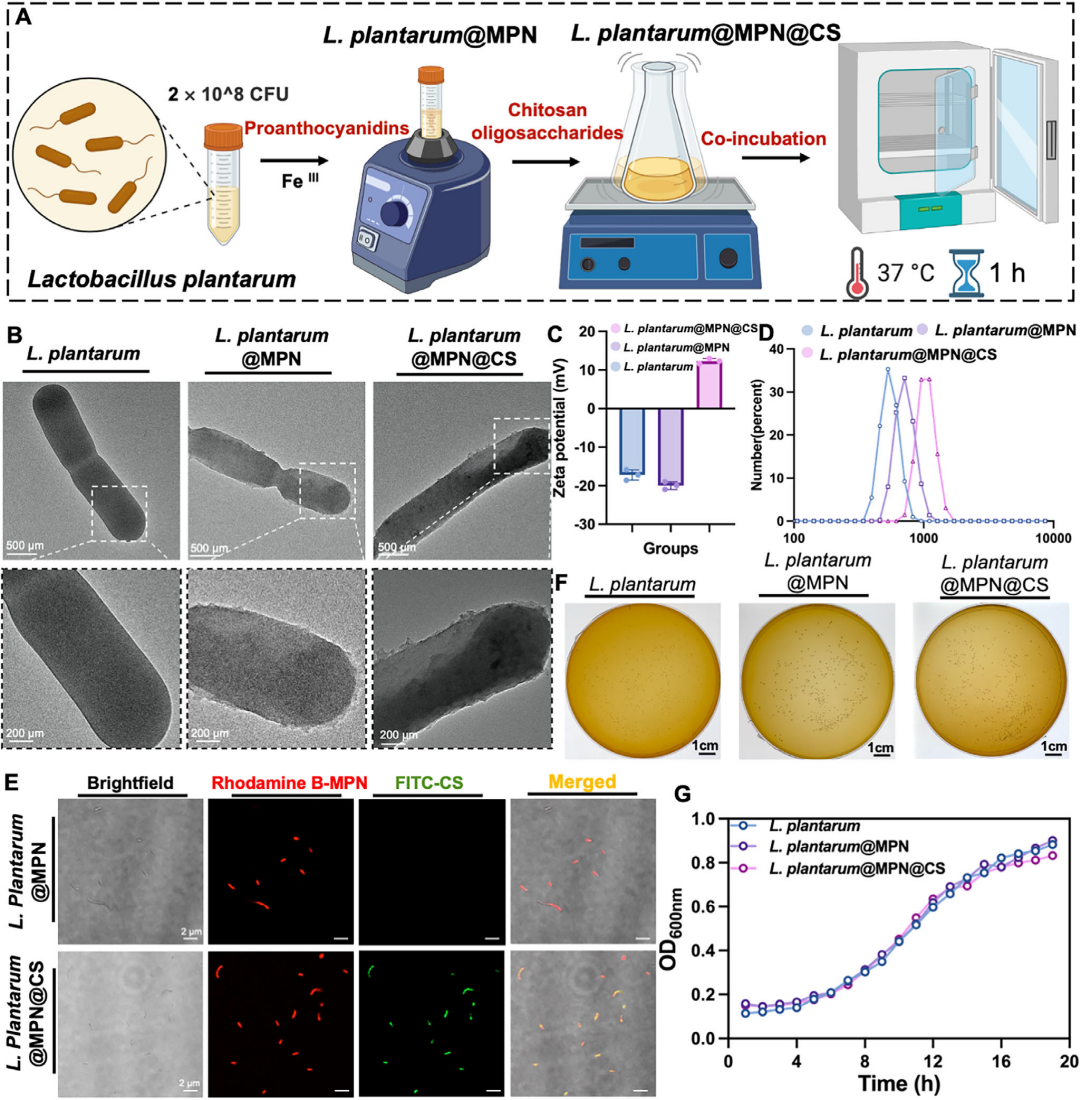
Preparation and characterization of *L. plantarum*@MPN@CS. (A) Diagram illustrating the synthesis of *L. plantarum*@MPN@CS, achieved through a layer‐by‐layer coating strategy involving the complexation of PC with Fe^III^ and subsequent integration of partially acetylated CS. The figure was created with BioRender.com. (B) Representative TEM images of native *L. plantarum*, *L. plantarum*@MPN, and *L. plantarum*@MPN@CS, respectively. Zeta potentials (C) and particle size distribution profiles (D) of *L. plantarum*, *L. plantarum*@MPN, and *L. plantarum*@MPN@CS, as determined by DLS (*n* = 3 for each group). (E) Typical confocal images of *L. plantarum*@MPN and *L. plantarum*@MPN@CS. The red channel corresponds to Rhodamine B‐labeled MPN layer, while the green channel visualizes FITC‐conjugated CS within the coatings. Scale bar: 2 µm. (F) Photographs of bacterial colonies formed on MRS agar plates of native *L. plantarum*, *L. plantarum*@MPN, and *L. plantarum*@MPN@CS. (G) Growth curves of *L. plantarum*, *L. plantarum*@MPN, and *L. plantarum*@MPN@CS in a MRS medium at 37°C, monitored by a microplate reader.

To enhance the therapeutic efficacy of *L. plantarum*, we developed a simple yet effective method for surface modification. In brief, polyphenols and ferric chloride (FeCl_3_) were sequentially mixed with *L. plantarum* cells to form MPN coatings. Initially, proanthocyanidins (PC) adheres to the bacterial surface via its catechol structures, subsequently undergoing cross‐linking through additional coordination with Fe^III^. A final layer of partially acetylated chitosan oligosaccharides (CS) was deposited on the PC‐Fe network, facilitated by multiple interactions between the phenolic hydroxyl and amino groups, resulting in a modified MPN nanoarmor (Figure 1A). The partially acetylated CS used in this study, with a deacetylation degree of 44%, was synthesized as previously described [33] (Figure S1). To visually confirm successful encapsulation, the morphology of single cells was examined via transmission electron microscopy (TEM). A clear continuous nanoshell with a thickness of ∼ 100 nm could be seen from *L. plantarum*@MPN@CS, contrasting with the smooth edges of the naked *L. plantarum* (Figure 1B). Additionally, particle size and zeta potential measurements further validated the stepwise coating process. The native *L. plantarum* exhibited an average particle size of 549.9 ± 86.94 nm and a zeta potential of ‐17.2 ± 1.35 mV. After modification, the particle size increased to 725.4 ± 123.3 nm for *L. plantarum*@MPN and further to 1051 ± 163.80 nm for *L. plantarum*@MPN@CS. Simultaneously, the zeta potential shifted from ‐20 ± 1.0 mV in *L. plantarum*@MPN to 12.3 ± 0.66 mV in *L. plantarum*@MPN@CS, confirming the successful coating (Figure 1C,D). These findings were further verified by the colocalization of Rhodamine B (RhB) marked‐MPN layer and FITC‐labeled CS layer fluorescence, as observed by laser scanning confocal microscopy (LSCM) (Figure 1E). Flow cytometric analysis was subsequently performed to assess labeling efficiency. The results demonstrated a significant shift of *L. plantarum*@MPN@CS to higher fluorescence intensities in both FITC and RhB channels, confirming successful decoration with both MPN and CS. Quantitative analysis of RhB^+^ FITC^+^ double‐positive *L. plantarum* indicated a labeling efficiency of over 75.7% (Figure S2). These findings collectively demonstrate the successful fabrication of the *L. plantarum*@MPN@CS.

Given the critical role of bacterial surface characteristics in mediating interactions with the surrounding environment, we evaluated the impact of the coatings on bacterial growth and viability. The growth curves were monitored over a 20 h period based on optical density at 600 nm (OD_600_), and no significant differences were observed between the coated and uncoated bacteria (Figure 1F,G), suggesting that the nanocoating had a negligible impact on the growth and viability of *L. plantarum*. However, some studies have reported that the coating layer can influence bacterial physiological functions, leading to a lag in growth or proliferation [23, 34, 35]. The discrepancies observed in these findings may arise from differences in coating thickness, the type of polyphenols employed, and the specific microbial species involved [12, 34]. These factors likely contribute to the differential effects on bacterial division and overall growth inhibition.

### 2.2 Enhanced Resistance Against Gastrointestinal Stresses In Vitro

**FIGURE 2:**
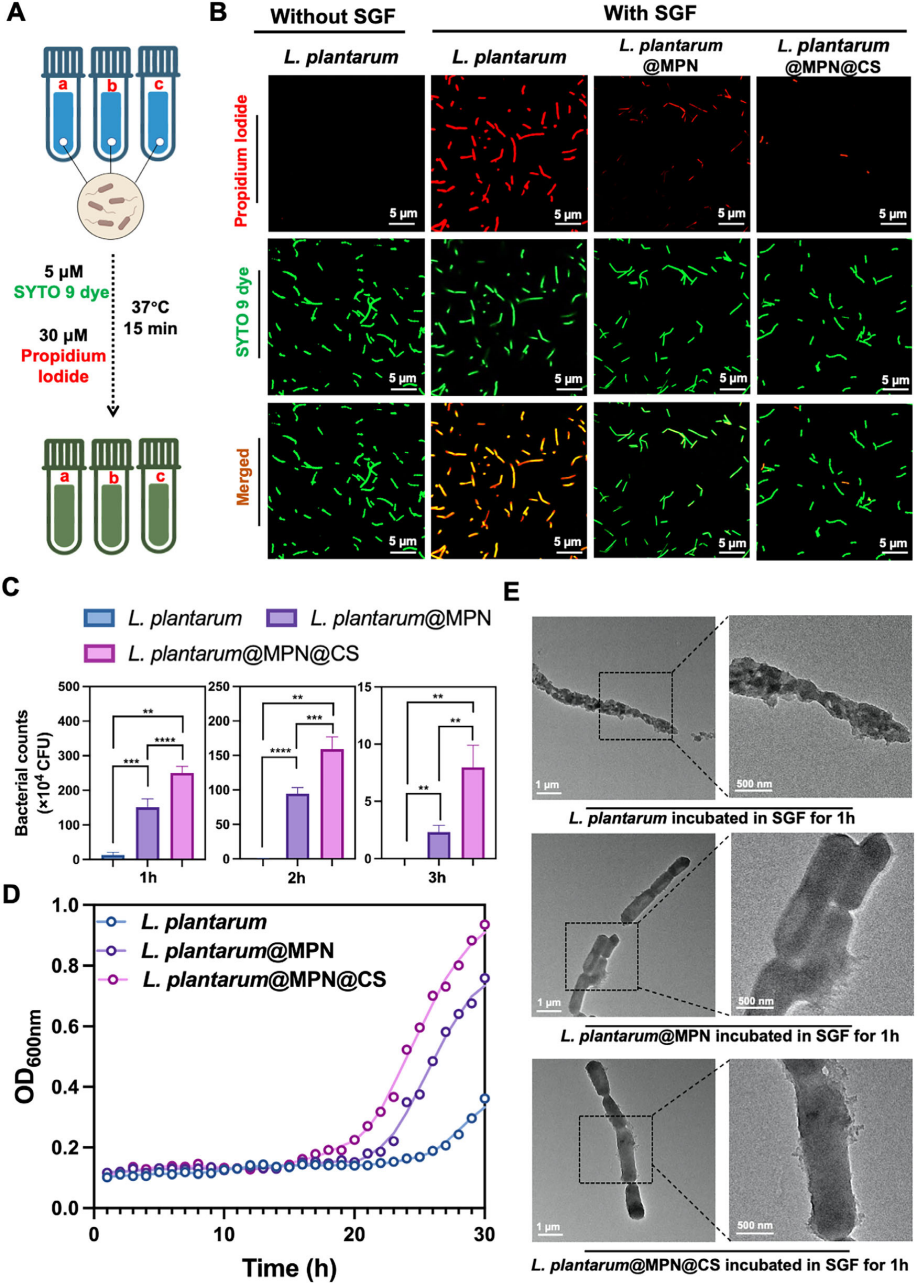
Resistance of *L. plantarum*@MPN@CS against gastrointestinal challenges (A) Diagram depicting the experimental design for assessing cell viability using live/dead staining. The figure was created with BioRender.com. (B) Representative confocal microscopy images of native *L. plantarum*, *L. plantarum*@MPN, and *L. plantarum*@MPN@CS, stained with SYTO 9 (green, indicating all bacteria) and PI (red, indicating dead bacteria), captured before and after a 1 h exposure to SGF. Scale bar: 5 µm. (C) Bacterial counts of naked and coated *L. plantarum* after exposure to SGF at different time points by plate counting. *n* = 3 for each group. (D) Growth curves of native *L. plantarum*, *L. plantarum*@MPN, and *L. plantarum*@MPN@CS cultured in MRS medium at 37°C following SGF treatment in vitro. (E) Representative TEM images of native *L. plantarum*, *L. plantarum*@MPN, and *L. plantarum*@MPN@CS after incubation in SGF (pH 2) 1 h at 37°C. Scale bar: 1 µm. Data are shown as mean ± SD. Statistical significance was determined by an unpaired, two‐tailed Student's *t*‐test. ^**^ *p* < 0.01, ^***^ *p* < 0.001, and ^****^ *p* < 0.0001.

Probiotics encounter various harsh conditions during transit through the GI tract, including gastric acid, bile salts, digestive enzymes, and ROS, all of which can destabilize and deactivate *L. plantarum* [11]. Therefore, we explored whether the nanoarmor could enhance the resistance of *L. plantarum* to these environmental insults in vitro. First, both naked and coated *L. plantarum* cells were exposed to simulated gastric fluid (SGF) supplemented with pepsin, mimicking the acidic gastric environment. Bacterial viability was assessed using the LIVE/DEAD BacLight Bacterial Viability Kit, which differentiates live and dead bacteria following incubation in SGF (Figure 2A). The results revealed that the acidic conditions of the gastric environment severely impaired the viability of *L. plantarum*, with nearly complete viability loss after 1 h of incubation. Conversely, the armored *L. plantarum*@MPN@CS exhibited significantly enhanced tolerance to SGF, achieving a higher survival rate under the same conditions (Figure 2B). To further assess the protective effect, cell viability of *L. plantarum* at different time points was measured using the plate counting method for *L. plantarum*, *L. plantarum*@MPN, and *L. plantarum*@MPN@CS. The results showed that *L. plantarum*@MPN@CS demonstrated considerably better tolerance to SGF, with a survival rate approximately 18.8‐fold higher than that of uncoated *L. plantarum* (Figure 2C). With the extension of the cultivation time to 3 h, a significant number of *L. plantarum*@MPN@CS cells still remained viable, while only a few *L. plantarum*@MPN cells survived, and no naked *L. plantarum* cells remained viable.

Subsequently, the samples were resuspended in MRS broth, and their OD_600_ values were measured over time. The results showed a significant increase in the growth activity of *L. plantarum*@MPN@CS as incubation time progressed, while the growth of *L. plantarum*@MPN and *L. plantarum* was delayed (Figure 2D). These findings confirm the better protective effects of the partially acetylated CS‐functionalized MPN nanocoating, as indicated by the superior growth tendency of *L. plantarum*@MPN@CS. TEM images in Figure 2E showed that the morphology of armored *L. plantarum*@MPN@CS and *L. plantarum*@MPN remained intact, with a partially detached coating shell, while naked *L. plantarum* exhibited aberrant morphology and complete damage upon prolonged incubation in SGF. These results highlight the critical role of nanocoating integrity and stability in shielding *L. plantarum* from gastric acid. Notably, *L. plantarum*@MPN@CS exhibited better protection than *L. plantarum*@MPN, which only had the PC‐Fe network. We speculate that the enhanced resistance to SGF may result from the capture of H^+^ ions from stomach acid by the amino groups in the partially acetylated CS, resulting in a protonation effect that reduces the surrounding acidity and mitigates the adverse effects of the acidic gastric environment. Moreover, the superior protective effects of the nanoarmor were further confirmed by the significantly improved survival of *L. plantarum*@MPN@CS after exposure to bile salts and simulated intestinal fluid (SIF) for the specified time points (Figures S3–S6).

Excessive ROS was one of the key pathological features associated with intestinal inflammation. The polyphenolic structure of PC has long been recognized for its potent antioxidant properties [36]. Therefore, we evaluated the ROS‐scavenging activity of *L. plantarum*@MPN@CS to examine its potential for oxidation resistance. Both ABTS and DPPH assays were used to measure the radical‐scavenging capabilities conferred by the nanoarmor. The results showed over 82.6% removal of the ABTS radical following treatment with *L. plantarum*@MPN@CS (Figure S7A). Similarly, the DPPH assay demonstrated an 88.0% clearance rate (Figure S7B). These findings confirm that the MPN layer exhibits excellent antioxidant properties, and that the inclusion of partially acetylated CS does not hinder the antioxidant activity of PC.

### 2.3 Prolonged Intestinal Retention of *L. plantarum*@MPN@CS

**FIGURE 3:**
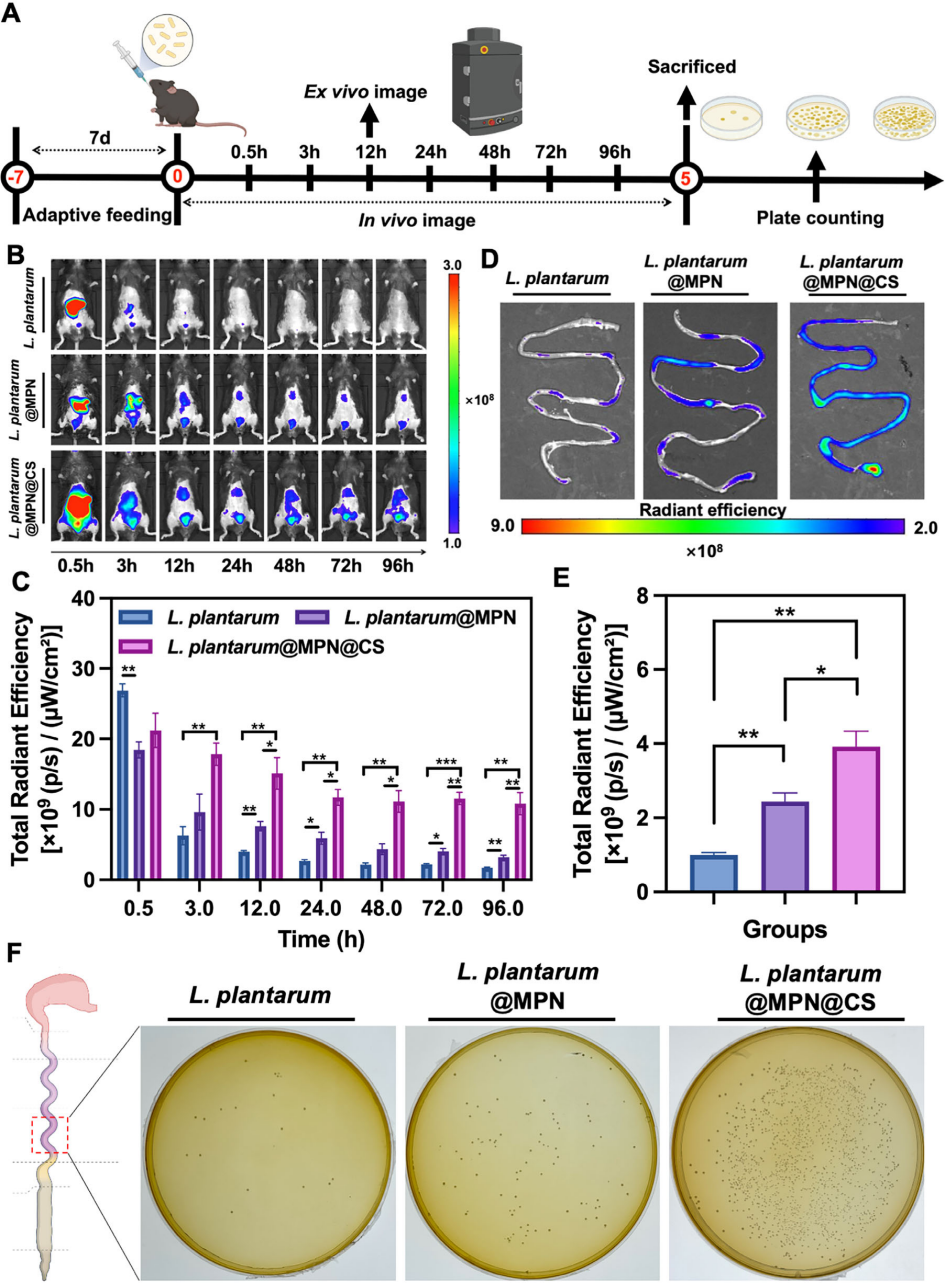
Biodistribution and adhesion of coated probiotics in vivo. (A) Schematic diagram of mouse intestinal biodistribution assay. The figure was created with BioRender.com. (B) Representative IVIS images of mice captured at 0.5, 4, 12, 24, 48, 72, and 96 h following the administration of different probiotic formulations labeled with pMG36e‐Px‐mCherry. (C) Total radiant efficiencies in the abdomen of mice treated with native *L. plantarum*, *L. plantarum*@MPN, and *L. plantarum*@MPN@CS following oral gavage at the indicated time points (*n* = 3 for each group). (D) IVIS images of mouse intestines at 12 h post‐administration of various probiotic formulations. (E) ROI analysis of intestinal tract fluorescence intensities at 12 h (*n* = 3 for each group). (F) Photographs of bacterial colonies in the ileum of mice treated with *L. plantarum*, *L. plantarum*@MPN, and *L. plantarum*@MPN@CS after 96 h of oral gavage. Data are presented as means ± SD. Statistical significance was determined by an unpaired, two‐tailed Student's *t*‐test. ^*^ *p* < 0.05, ^**^ *p* < 0.01, and ^***^ *p* < 0.001.

The limited retention time of probiotics within the GI tract remains another significant barrier to their therapeutic efficacy [37]. Therefore, the ability of probiotics to adhere to intestinal mucus post‐gastric transit is crucial for optimizing their physiological functions. Encouraged by the aforementioned in vitro superior gastrointestinal stress resistance ability of nanocoating‐armored *L. plantarum*, we therefore investigated the biodistribution and adhesion of *L. plantarum*, *L. plantarum*@MPN, and *L. plantarum*@MPN@CS in the GI tract under physiological conditions. To achieve this goal, *L. plantarum* strains were electroporated with the fluorescent reporter plasmid pMG36e‐Px‐mCherry (*L. plantarum*‐mCherry) for in vivo fluorescence imaging. Equal amounts (1 × 10^8 CFU) of uncoated and coated *L. plantarum*‐mCherry were orally administered to mice, and the biodistribution was monitored over time using an in vivo imaging system (IVIS) (Figure 3A). As shown in Figure 3B, the fluorescence intensity in the abdomen of mice treated with uncoated *L. plantarum*‐mCherry significantly decreased within 3 h post‐administration, indicating poor gut retention. In contrast, *L. plantarum*‐mCherry@MPN and *L. plantarum*‐mCherry@MPN@CS exhibited sustained fluorescence, detectable even after 96 h, demonstrating enhanced intestinal retention. Notably, *L. plantarum*‐mCherry@MPN@CS showed higher fluorescence intensity after 12 h post‐gavage compared to both *L. plantarum*‐mCherry and *L. plantarum*‐mCherry@MPN (Figure 3B,C).

To further assess retention, the GI tract was harvested 12 h post‐administration for ex vivo IVIS imaging. As illustrated in Figure S8, the fluorescence signal of *L. plantarum* was predominantly observed in the stomach, small intestine (jejunum and ileum), and cecum, with the highest intensity detected in the small intestine. This finding confirms that the small intestine was the primary site of *L. plantarum* accumulation. This distribution pattern suggests a preferential colonization of the small intestine by *L. plantarum*, consistent with previous studies [38]. Notably, both coated formulations displayed markedly increased accumulation in the small intestine compared with uncoated bacteria, with *L. plantarum*‐mCherry@MPN@CS showing the strongest fluorescence (Figure 3D,E). Moreover, quantification of viable *L. plantarum*‐mCherry cells in the ileum (distal small intestine) revealed a 12.3‐fold increase in the number of viable cells for *L. plantarum*‐mCherry@MPN@CS compared to *L. plantarum*‐mCherry@MPN and a 45.4‐fold increase compared to uncoated *L. plantarum*‐mCherry (Figure 3F; Figure S9). These findings demonstrate that the nanocoating greatly enhances survival and colonization efficiency in healthy mice. The underlying mechanism is attributed to the PC‐Fe network and partially acetylated CS layers, which interact with mucus to prolong *L. plantarum* retention and support sustained probiotic activity.

Mucosal adhesion is a key factor for prolonging the retention time of probiotics in the GI tract, thereby enhancing their colonization and therapeutic potential [9]. Previous studies have demonstrated that MPNs exhibit robust mucosal adhesion via hydrogen bonding, covalent bonding, electrostatic interactions, and *π–π* stacking between catechol groups and mucin [39]. The addition of a cationic, partially acetylated CS layer enhances electrostatic interactions with the negatively charged mucus layer, further promoting adhesion. Functional groups on CS molecules—including amino and acetylamino groups—enable additional interactions with mucin via hydrogen bonding, hydrophobic forces, and electrostatic attraction. These synergistic interactions significantly improve the adhesion of *L. plantarum* to the intestinal mucosa, leading to enhanced colonization and prolonged retention in the gut.

### 2.4 Preferential Adhesion and Enhanced Colonization Under Physiological Conditions

**FIGURE 4:**
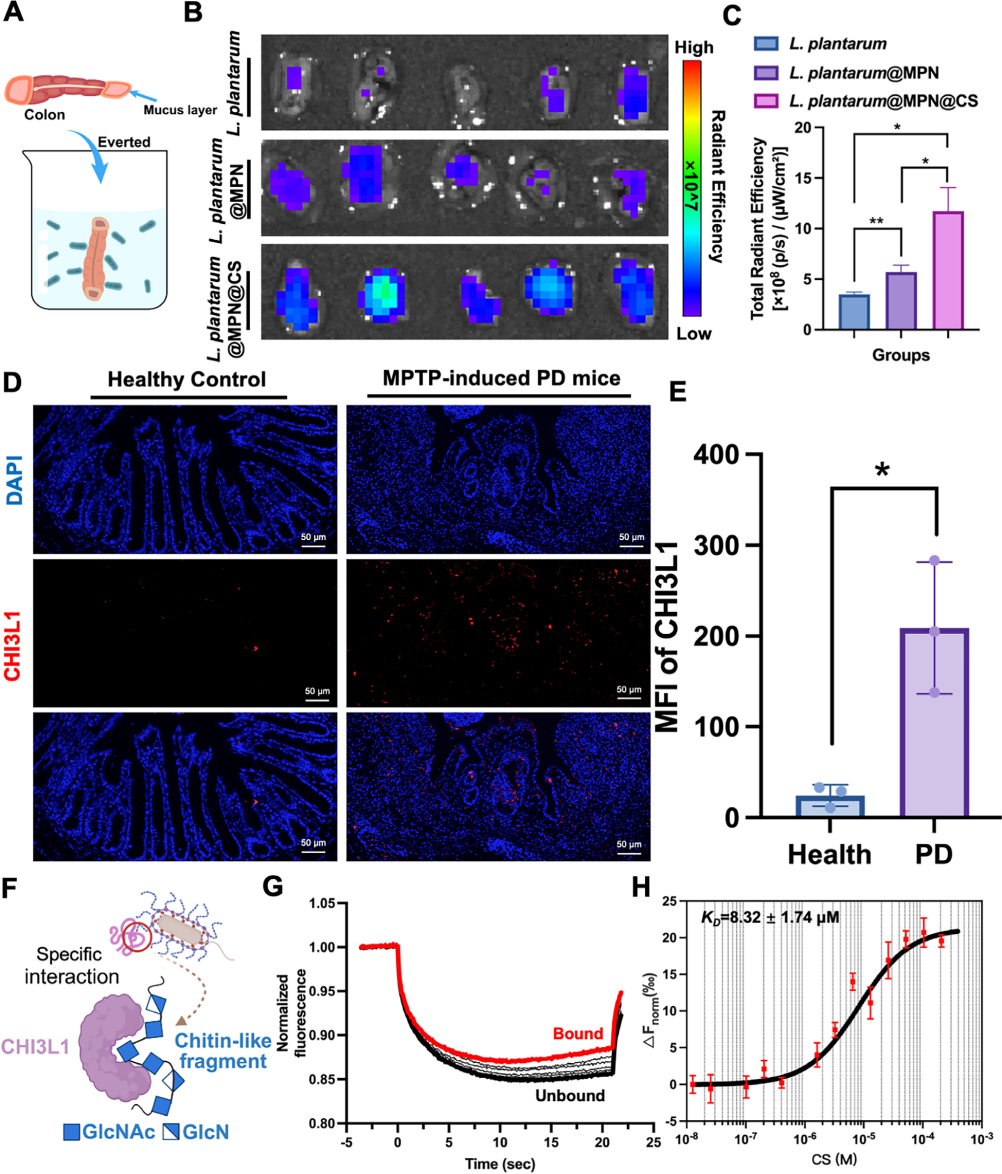
Targeted delivery and enhanced retention of *L. plantarum*@MPN@CS in the PD‐related pathological intestine. (A) Schematic illustration of experimental design for examining the reaction efficiency between *L. plantarum*@MPN@CS and the intestine of PD mice. Representative IVIS images (B) and corresponding fluorescence intensities (C) of everted murine intestine segments after cultivation with equivalent naked *L. plantarum* or coated *L. plantarum* expressing mCherry protein. *n* = 5 for each group. (D) Immunofluorescent staining with anti‐CHI3L1 antibody (red) on the intestine harvested from healthy and PD mice. DAPI was used to stain the nucleus. Scale bar: 50 µm. (E) Semiquantitative analysis of the expression of CHI3L1 based on immunofluorescence staining (*n* = 3 for each group). (F) Diagram illustrating the specific interaction between CHI3L1 and partially acetylated CS containing chitin‐like fragment. The figure was created with BioRender.com. (G) Relative fluorescence intensity between the bound and unbound states. The thermophoretic movement of a fluorescently labeled molecule (black trace; “unbound”) changes upon binding to a non‐fluorescent ligand (red trace; “bound”), resulting in distinct traces. (H) A typical MST curve for the interaction of partially acetylated CS with CHI3L1. A *K_D_* of 8.32 ± 1.74 µm was determined for this interaction using standard data analysis with MO Affinity Analysis Software. The graphs display data from three independent measurements (*n* = 3). Data are presented as means ± SD. Statistical significance was determined by an unpaired, two‐tailed Student's *t*‐test. ^*^ *p* < 0.05 and ^**^ *p* < 0.01.

Orally administered live biotherapeutics benefit from prolonged retention at the lesion site, but the pathological inflammatory microenvironment of diseases poses challenges to probiotic retention [12]. Functionalized nanoarmors that address adhesion and colonization in dynamic, disease‐affected gastrointestinal environments are critical for effective therapeutic modulation. We next investigate the ability of our designed microenvironment self‐adaptive nanoarmor to enhance the accumulation of *L. plantarum* in the PD‐related pathological conditions. The intestines of PD mice were collected, everted, and incubated with naked *L. plantarum*‐mCherry, *L. plantarum*‐mCherry@MPN, and *L. plantarum‐mCherry*@MPN@CS (Figure 4A). After 1 h of incubation, fluorescence imaging via IVIS revealed that *L. plantarum*‐mCherry@MPN@CS‐treated intestinal segments exhibited significantly higher fluorescence intensity than both the naked *L. plantarum*‐mCherry and *L. plantarum*‐mCherry@MPN groups (Figure 4B), indicating superior retention in the PD‐related pathological intestine. Quantitative analysis confirmed a 3.3‐fold increase in bacterial retention in the *L. plantarum*‐mCherry@MPN@CS group compared to the naked *L. plantarum*‐mCherry group (Figure 4C). Notably, the *L. plantarum*‐mCherry@MPN group also showed higher fluorescence intensity than the naked *L. plantarum*‐mCherry group, likely due to the more negatively charged MPN coating enhancing retention through electrostatic interactions with cation‐rich inflammatory lesions [40, 41].

To further evaluate the role of partially acetylated CS in prolonged retention at the inflammatory site, polyethylenimine (PEI) was used to prepare *L. plantarum*‐mCherry@MPN@PEI, resulting in a comparable zeta potential to the CS‐coated bacteria (Figure S10A). However, the *L. plantarum*‐mCherry@MPN@CS group exhibited significantly higher fluorescence intensity than both *L. plantarum*‐mCherry and *L. plantarum*‐mCherry@MPN@PEI groups, highlighting the superior retention afforded by partially acetylated CS in the PD‐related inflammatory environment (Figure S10B). Conversely, the *L. plantarum*‐mCherry@MPN@PEI group showed slightly lower fluorescence intensity than the naked *L. plantarum* group, suggesting that only the positive surface charge of PEI may be less effective in the mucus‐depleted PD gut, where surface charge reversal occurs. These results demonstrate that *L. plantarum*‐mCherry@MPN@CS preferentially localizes to the PD‐related GI tract, demonstrating the ability of nanoarmor to overcome adhesion‐ and colonization‐related obstacles in disease‐affected gastrointestinal environments.

Chitinase‐3‐Like Protein 1 (CHI3L1) is an inflammatory biomarker upregulated in various disease‐related conditions [27]. Although classified within the glycoside hydrolase family, CHI3L1 lacks catalytic activity and binds to chitin through a carbohydrate‐binding motif [28]. We observed that CHI3L1 levels were elevated in PD mice intestinal tissues (Figure 4D) and increased approximately 8.6‐fold compared to healthy controls (Figure 4E). This suggests that CHI3L1 may act as a colonization niche, promoting bacterial retention through its high‐affinity binding to partially acetylated CS layer. To assess this hypothesis, we used microscale thermophoresis (MST) to evaluate the binding affinity of partially acetylated CS with CHI3L1. Binding analysis revealed a strong affinity, with a *K_D_* value of 8.32 ± 1.74 µm (Figure 4F–H), which is consistent with previous studies showing a strong interaction between CHI3L1 and chitin‐like molecules [26, 42]. In the PD gut, CHI3L1 likely interacts with partially acetylated CS‐coated bacteria, promoting bacterial colonization in the inflammatory sites. To determine the specificity of this interaction, we compared partially acetylated CS binding with transferrin (Tf), another abundant secretory protein in the inflammatory gut [12]. While Tf expression was significantly increased (∼6.2‐fold) in the PD gut (Figure S11), MST analysis showed weaker binding between partially acetylated CS and Tf, further supporting the preferential interaction between partially acetylated CS and CHI3L1 in the PD gut milieu rather than to other highly abundant secretory proteins (Figure S12). These findings highlight the ability of *L. plantarum*‐mCherry@MPN@CS to enhance probiotic retention and colonization in the PD‐related pathological microenvironment. The preferential interaction with CHI3L1 in the pathological gut demonstrates the potential of partially acetylated CS functionalized nanoarmor to address adhesion and colonization obstacles in disease‐affected gastrointestinal environments.

### 2.5 *L. plantarum*@MPN@CS Improves Motor Dysfunction of PD Mice

**FIGURE 5:**
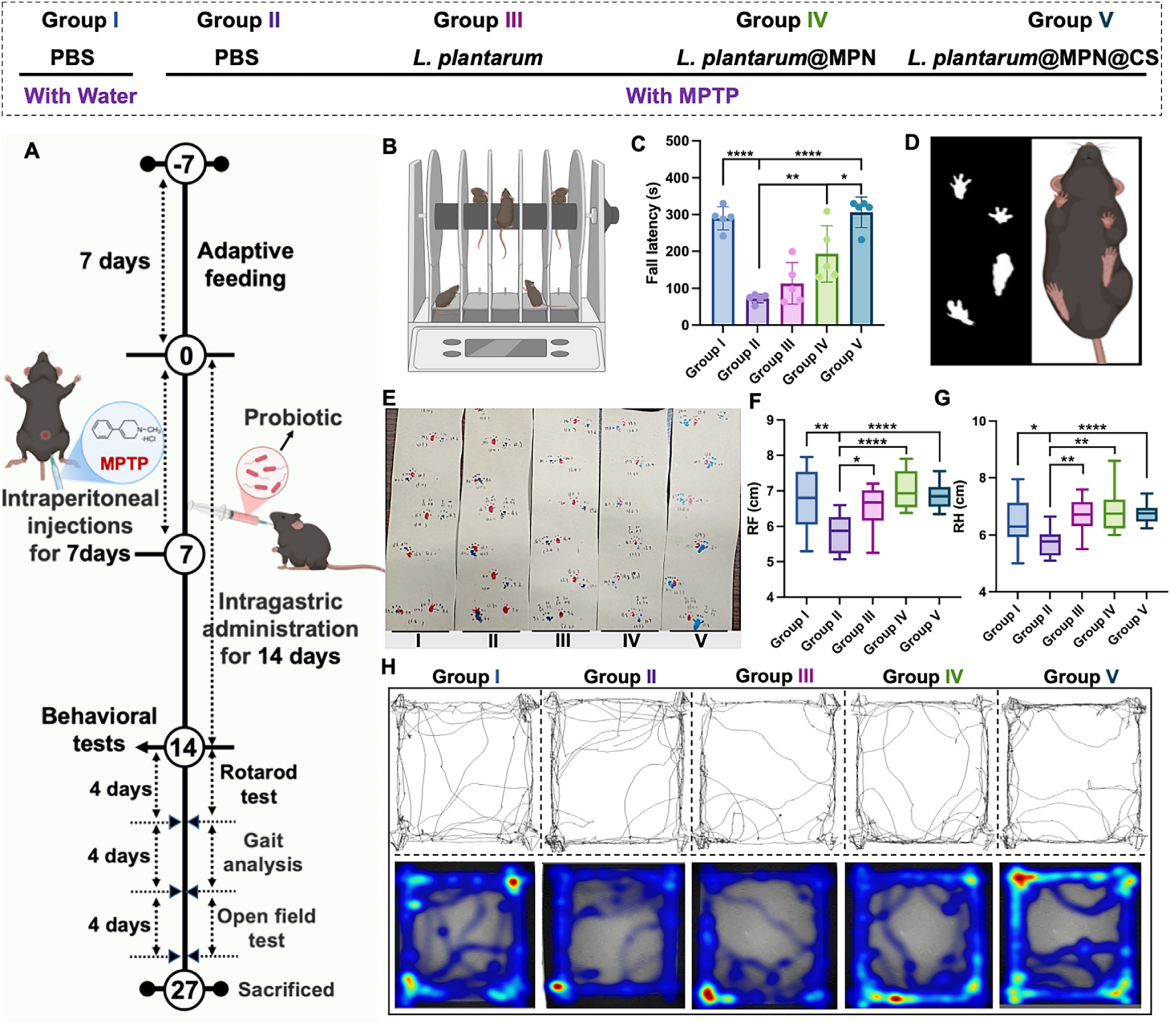
*L. plantarum*@MPN@CS ameliorates motor impairments in an MPTP‐induced subacute PD model. (A) Experimental design for evaluating the therapeutic effects of *L. plantarum*@MPN@CS. The figure was created with BioRender.com. (B) Schematic diagram of the rotarod test. The figure was created with BioRender.com. (C) Time taken for mice administered with various probiotic formulations to fall from the rod (*n* = 5 per group). (D) Schematic illustration of the gait analysis. (E) Representative images of the footprint on paper. (F) Stride lengths measured for the right forelimb (RF) and (G) right hindlimb (RH) in each group (*n* = 5 per group). At least four footprints from each mouse were used for data analysis, with the initial footprints excluded from statistical analysis, as the mice may not have yet entered a continuous walking phase. (H) Representative traced movement paths and typical tracking motion heatmap from the open field test. Data are presented as means ± SD. Statistical significance was determined by an unpaired, two‐tailed Student's *t*‐test. ^*^ *p* < 0.05, ^**^ *p* < 0.01, and ^****^ *p* < 0.0001.

Building upon the confirmed enhanced survival and retention of coated bacteria, and the promising results for overcoming adhesion and colonization challenges in disease‐affected gastrointestinal environments, we evaluated whether *L. plantarum*@MPN@CS could serve as an effective therapeutic for MPTP‐induced subacute PD in C57BL/6J mice. Experimental details are shown in Figure 5A. C57BL/6J mice were randomly assigned to five groups: PBS group (healthy control, Group I), PD group (disease model without treatment, Group II), PD + *L. plantarum* group (Group III), PD + *L. plantarum*@MPN group (Group IV), and PD + *L. plantarum*@MPN@CS group (Group V). Throughout the experimental period, mice received either PBS or bacterial formulations (5 × 10^8 CFU) via oral gavage based on their group assignment. Meanwhile, MPTP (30 mg/kg) was injected intraperitoneally to induce PD, except in the PBS group. Seven days post‐injection, mice were subsequently treated with their respective formulations for an additional 7 days.

Motor dysfunction is one of the important characteristics of PD, which reflects dopamine depletion [43]. Representative behavioral tests (including the rotameter test, gait analysis, and open field test) were conducted following the protocol outlined in a previously reported user's guide to assess motor dysfunction resulting from dopaminergic damage in the mouse brain [44]. To assess the motor coordination, the residence time of mice on a rotating rod was measured by rotarod tests (Figure 5B). Mice treated with coated bacteria (*L. plantarum*@MPN and *L. plantarum*@MPN@CS) showed significantly increased residence times than the untreated PD group (Figure 5C). Notably, the *L. plantarum*@MPN@CS group showed superior performance compared to the *L. plantarum*@MPN group. Gait analysis revealed no significant differences in claw length, claw width, or average footprint area across the groups (Figure 5D,E). However, both treated groups exhibited longer stride lengths in the right forelimb and right hindlimb compared to the MPTP group, with the *L. plantarum*@MPN@CS treated group showing the most improvement (Figure 5F,G; Figure S13). This interlimb asymmetry and gait shortening observed in Parkinson's patients are linked to basal ganglia‐mediated motor control, particularly involving dopaminergic neurons in the substantia nigra pars compacta [45]. Moreover, the open field test was used to evaluate the spontaneous motor activity of mice (Figure 5H). MPTP injection significantly reduced the locomotor activity, as indicated by reduced walking distance and lower average speed (Figure S14). In contrast, the total traveled distance and average speed were significantly increased in PD mice, especially after treatment with coated *L. plantarum* formulations, indicating improvement in locomotor function. The limited therapeutic efficacy of naked *L. plantarum* can be attributed to its susceptibility to environmental insults, reduced colonization capacity, and inadequate retention under physiological conditions, limiting its application as an oral live biotherapeutic. Notably, *L. plantarum*@MPN@CS exhibited significantly superior efficacy compared to *L. plantarum*@MPN. The superior performance of *L. plantarum*@MPN@CS is due to the unique features of partially acetylated CS, which provides a tunable ratio of GlcN and GlcNAc, allowing the formulation to adapt its interactions within complex physiological and pathological microenvironments, thereby enhancing its therapeutic potential.

In conclusion, *L. plantarum*@MPN@CS treatment demonstrated superior efficacy in improving motor dysfunction in MPTP‐induced PD mice, outperforming both *L. plantarum* and *L. plantarum*@MPN formulations. The enhanced therapeutic effects of *L. plantarum*@MPN@CS are attributed to its resilience in harsh gastrointestinal conditions, improved intestinal adhesion, and sustained colonization. Notably, the partially acetylated CS‐functionalized nanoarmor addresses key adhesion and colonization challenges in disease‐affected gastrointestinal environments, which is critical for its therapeutic success in PD treatment.

### 2.6 *L. plantarum*@MPN@CS Attenuated Dopaminergic Neuronal Death and Reduced Neuroimmune Response

**FIGURE 6:**
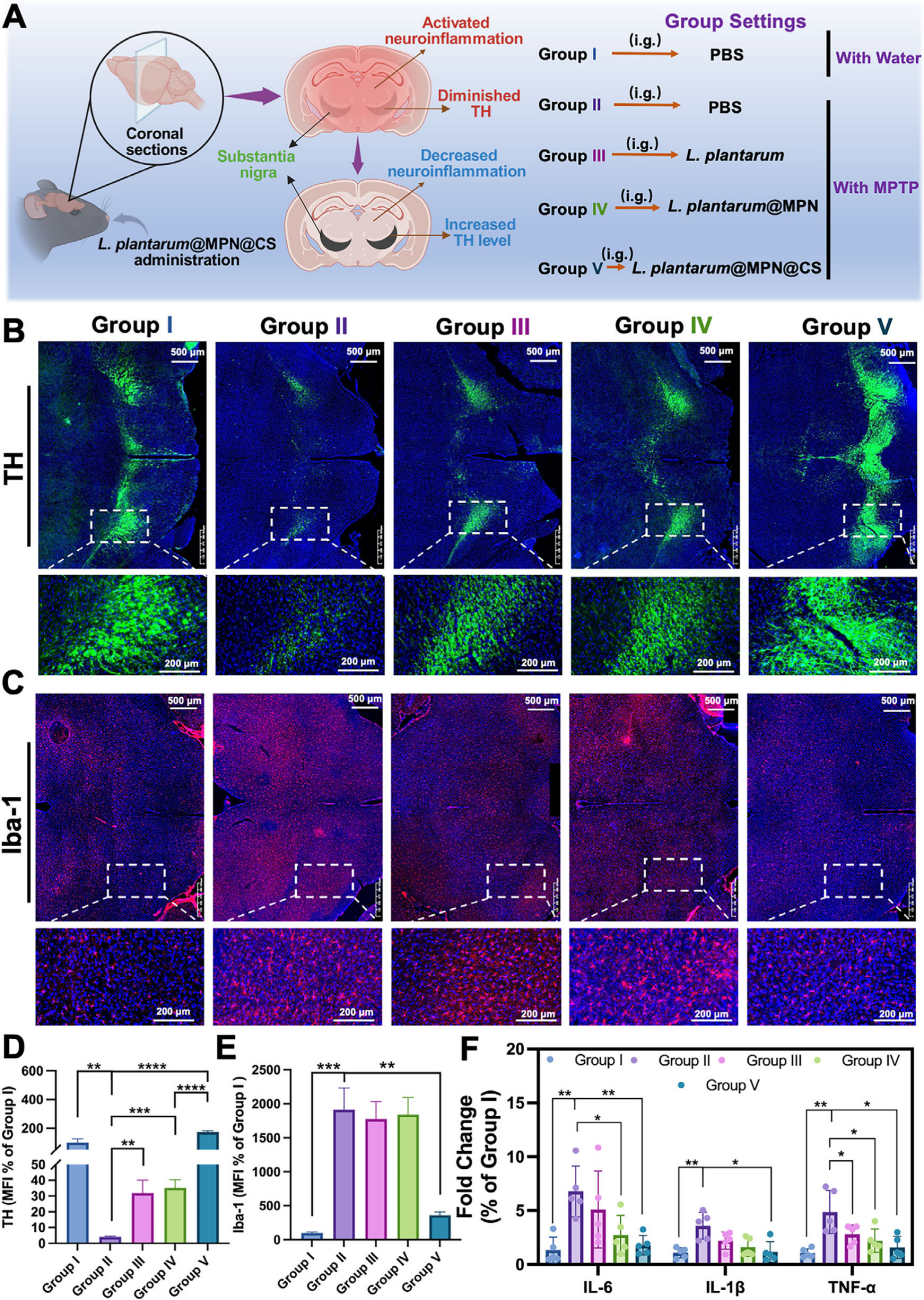
Alleviation of central inflammation and restoration of dopaminergic neurons. (A) Schematic illustration and group settings of the experimental design used to assess the therapeutic efficacy of *L. plantarum*@MPN@CS in restoring brain functional deficits. The figure was created with BioRender.com. (B) Representative immunofluorescence images showing dopaminergic neurons in the substantia nigra from different groups. Green fluorescence represents TH‐marked dopaminergic neurons, and DAPI was used for nuclear staining. Scale bar: 500 µm. (C) Representative immunofluorescence images of Iba‐1 (red) with DAPI (blue) counterstaining. Scale bar: 500 µm. (D) Semi‐quantitative analysis of TH expression and (E) Iba‐1 expression in the brain, based on immunofluorescence staining (*n* = 3 per group). (F) RT‐qPCR analysis of pro‐inflammatory cytokines in brain tissue homogenates from different groups (*n* = 5 per group). Data are presented as means ± SD. Statistical significance was determined by an unpaired, two‐tailed Student's *t*‐test. ^*^ *p* < 0.05, ^**^ *p* < 0.01, ^***^ *p* < 0.001, and ^****^ *p* < 0.0001.

The reduction of tyrosine hydroxylase (TH) levels in brain tissues is a hallmark of PD, as TH serves as the rate‐limiting enzyme in dopamine synthesis, playing a crucial role in dopaminergic neuronal function [46]. To further assess the therapeutic efficacy of the treatments, we evaluated TH expression in the substantia nigra (SN) and striatum (Figure 6A; Figure S15). Immunofluorescence staining revealed a significant loss of TH‐positive cells in the substantia nigra (SN) of MPTP‐treated mice (Figure 6B), indicating a marked reduction in dopaminergic neurons compared to healthy controls. As expected, mice treated with bacterial formulations showed significantly higher TH levels compared to those receiving PBS (Figure 6B; Figure S16). Notably, the *L. plantarum*@MPN@CS treatment demonstrated a superior ability to mitigate MPTP‐induced neurotoxicity compared to other groups. Quantification of relative fluorescence intensity showed that TH expression in treated mice reached 31.9% (Group III), 35.2% (Group IV), and 172.5% (Group V) of the control levels (Figure 6D), respectively, suggesting the neuroprotective potential of these treatments. A similar increase in the number of TH‐positive neurons was observed in the striatum, with the *L. plantarum*@MPN@CS‐treated group showing the highest fluorescence intensity (Figures S17 and S18).

Elevated levels of pro‐inflammatory factors and activated microglia are common in the pathophysiology of PD, contributing to neuroinflammation that exacerbates dopaminergic neuron death and accelerates disease progression [47]. To elucidate the potential mechanisms underlying the neuroprotective effects of *L. plantarum*@MPN@CS treatment in the PD model, we evaluated neuroinflammation in the mouse brain. Microglial activation, a direct manifestation of neuroinflammation, can be reflected by upregulation of Iba‐1 (an M1 microglial marker) [48]. Iba‐1 immunofluorescence staining revealed extensive microglial activation in the SN of MPTP‐treated mice, characterized by a high density of Iba‐1‐positive cells (Figure 6C; Figure S19). In contrast, treatment with *L. plantarum*@MPN@CS significantly reduced Iba‐1 expression, suggesting a decrease in microglial activation (Figure 6C,E; Figure S19). This reduction in activation was further corroborated by a significant decrease in the levels of pro‐inflammatory cytokines IL‐6, IL‐1β, and TNF‐α in brain tissue (Figure 6F), indicating the anti‐inflammatory effects of *L. plantarum*@MPN@CS treatment. In comparison, *L. plantarum* alone displayed minimal therapeutic efficacy in reducing microglial activation. Collectively, these results suggest that *L. plantarum*@MPN@CS may have therapeutic potential in ameliorating PD progression by attenuating dopaminergic neuronal death and modulating the neuroimmune response.

### 2.7 *L. plantarum*@MPN@CS Enhanced Gut Barrier Integrity and Alleviated Peripheral Inflammation

The gut‐brain axis plays a crucial role in various neurological conditions, including PD. This bidirectional communication network between the gut and brain is particularly relevant in PD, where gastrointestinal dysfunction is a hallmark feature [49]. Dysbiosis and compromised intestinal permeability are important contributors to PD pathogenesis [50, 51]. To investigate the potential therapeutic effects of *L. plantarum*@MPN@CS in PD, we assessed intestinal barrier integrity as a potential rescue mechanism in an MPTP‐induced PD mouse model.

**FIGURE 7:**
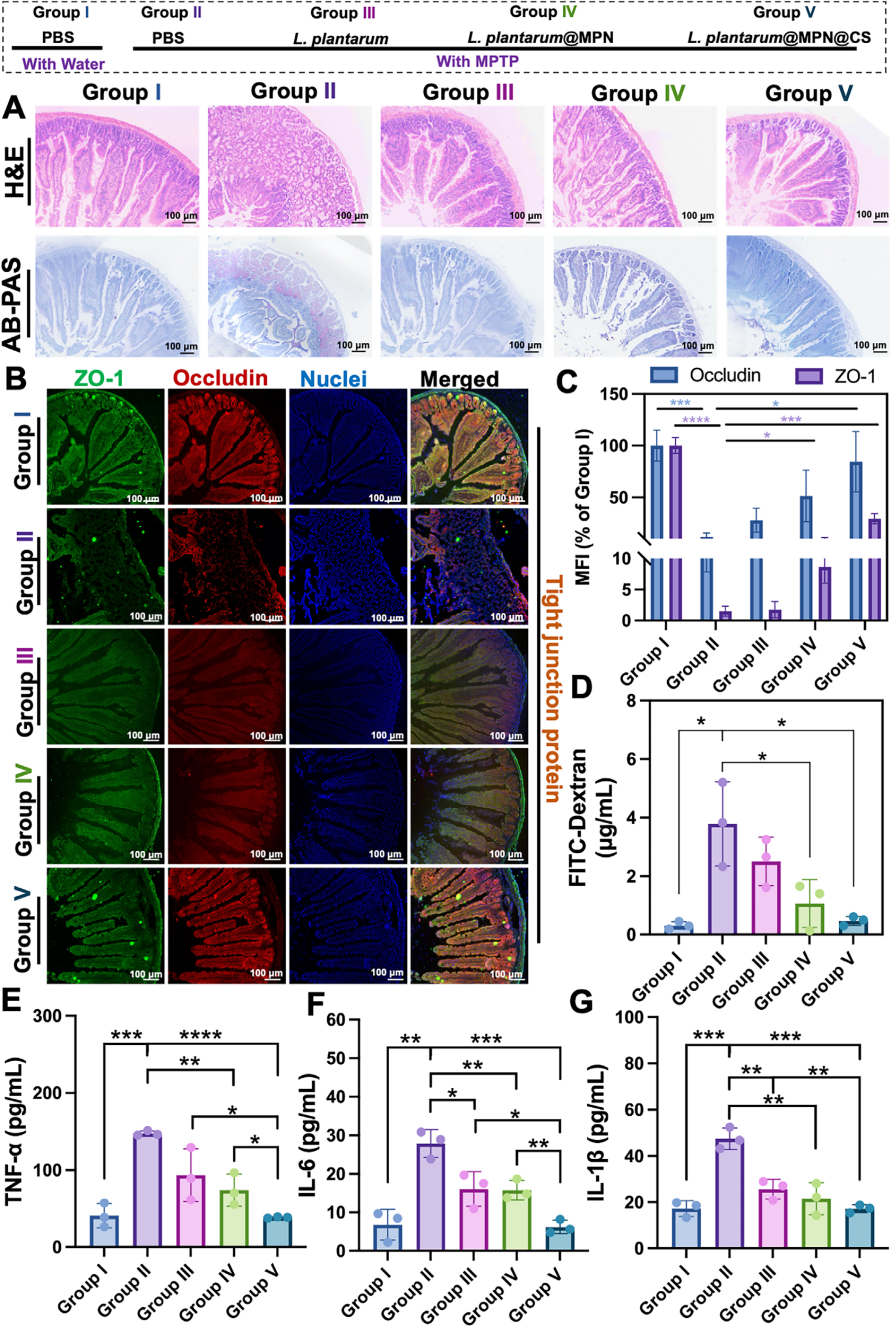
*L. plantarum@MPN@CS* protects the intestinal barrier from damage and reduces peripheral inflammation in PD mice. (A) Representative histological images of colon tissues stained with hematoxylin and eosin (H&E) and alcian blue‐periodic acid‐ schiff (AB‐PAS). (B) Immunofluorescence staining of ZO‐1 and occludin in the intestine (green: ZO‐1, red: occludin, blue: nuclei). Scale bar: 100 µm. (C) Semi‐quantitative analysis of occludin and ZO‐1 expression based on immunofluorescence staining (*n* = 3 for each group). (D) Intestinal barrier functions assessed by in vivo intestinal permeability assays (*n* = 3 per group). (E) Production of pro‐inflammatory cytokines TNF‐α, (F) IL‐6, and (G) IL‐1β in the serum of mice from different groups (*n* = 3 per group). Data are presented as means ± SD. Statistical significance was determined by an unpaired, two‐tailed Student's *t*‐test. ^*^ *p* < 0.05, ^**^ *p* < 0.01, ^***^ *p* < 0.001, and ^****^ *p* < 0.0001.

Histological analysis, including H&E and AB‐PAS staining (Figure 7A), revealed significant damage to the intestinal barrier in MPTP‐treated PD mice, characterized by epithelial cell damage and inflammatory cell infiltration. In contrast, *L. plantarum*@MPN@CS treatment significantly reduced the inflammatory response, preserved the villus structure, and restored epithelial integrity. AB‐PAS staining further highlighted the protective effects of treatment on the mucosal layer, with a notable reduction in mucosal loss in PD mice. To assess the impact of *L. plantarum*@MPN@CS on intestinal permeability, we performed immunofluorescence staining of key tight junction proteins, ZO‐1 and occludin, which are essential for maintaining intestinal barrier function [52]. In MPTP‐induced PD mice, the fluorescence intensity of both ZO‐1 and occludin was significantly reduced, indicating disrupted barrier integrity. However, treatment with *L. plantarum*@MPN@CS markedly increased the fluorescence intensity of both proteins, suggesting a restoration of intestinal barrier function (Figure 7B,C). Further, to quantify the restoration of intestinal permeability, we conducted in vivo assays using FITC‐dextran (4 kDa) as a tracer [53]. After a 4 h fasting period, mice were gavaged with FITC‐dextran (0.6 g/kg body weight), and serum levels of FITC‐dextran were measured after 6 h. The results demonstrated that *L. plantarum*@MPN@CS treatment significantly reduced systemic exposure to FITC‐dextran in MPTP‐treated PD mice (Figure 7D), compared to the PBS, *L. plantarum*, and *L. plantarum*@MPN groups, further supporting the restoration of intestinal barrier integrity.

Increased intestinal permeability in PD is known to facilitate bacterial translocation, which can trigger systemic inflammation and disrupt the blood‐brain barrier [54]. This process may lead to microglial activation and the release of pro‐inflammatory cytokines in the central nervous system, contributing to neuroinflammation. To investigate these systemic inflammatory responses, we analyzed serum levels of TNF‐α (Figure 7E), IL‐6 (Figure 7F), and IL‐1β (Figure 7G). MPTP‐treated PD mice exhibited significantly elevated concentrations of these pro‐inflammatory cytokines compared to healthy controls. Notably, treatment with *L. plantarum*@MPN@CS led to a substantial reduction in these cytokine levels, demonstrating its ability to restore intestinal barrier integrity and attenuate peripheral inflammation. These findings were further corroborated by a decrease in the expression of pro‐inflammatory cytokines in the brain and a reduction in the number of activated microglia, as shown in Figure 6.

Together, our findings provide compelling evidence that *L. plantarum*@MPN@CS restores intestinal barrier integrity in PD by enhancing the expression of tight junction proteins and reducing intestinal permeability. This restoration not only alleviates systemic inflammation but also mitigates neuroinflammatory processes in the brain. These results underscore the therapeutic potential of *L. plantarum*@MPN@CS in modulating the gut‐brain axis as a strategy for managing PD.

### 2.8 *L. plantarum*@MPN@CS Altered Gut Microbiota

Accumulating evidence underscores the pivotal role of microbial dysbiosis in the onset and progression of PD [55]. The composition of the gut microbiota is significantly altered in PD patients compared to healthy individuals, often leading to a disruption of gut‐brain axis communication [56]. To explore whether *L. plantarum*@MPN@CS could reshape the dysbiosis of gut microbiota in PD mice and potentially halt disease progression, we conducted a comprehensive analysis using 16S rDNA sequencing.

**FIGURE 8:**
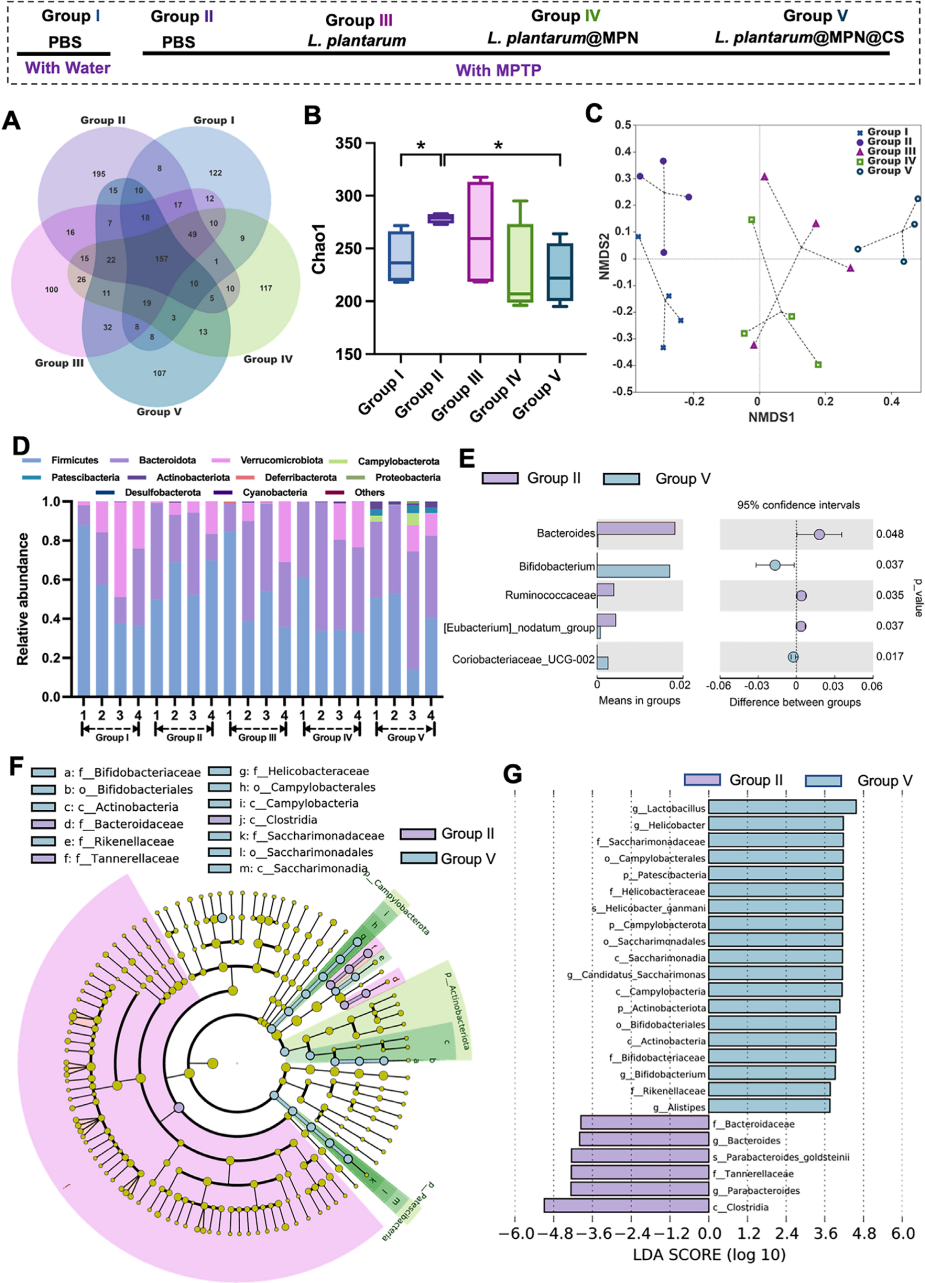
*L. plantarum*@MPN@CS modulates the composition of gut microbiota during PD treatment. (A) Venn diagram based on amplicon sequence variants (ASVs). (B) Comparison of alpha diversity assessed by the Chao1 index. (C) Grouping of gut microbial communities across different cohorts as depicted in the NMDS plot based on Bray‐Curtis distance. (D) Column diagram of the relative abundance of gut microbiome at the phylum level. (E) Comparison of enriched bacterial taxa at the family level between the PD group and the group administered with *L. plantarum*@MPN@CS. (F) Cladogram based on Linear discriminant analysis effect size (LEfSe) analysis showing community composition of the gut microbiota in mice. (G) Distribution histogram based on linear discriminant analysis (LDA). An LDA score higher than 3 indicates a higher relative abundance in the corresponding group than in other groups. LDA (log10) > 3.0, *p* < 0.05. Data are presented as means ± SD. Statistical significance was determined by an unpaired, two‐tailed Student's *t*‐test. ^*^ *p* < 0.05.

As illustrated in Figure 8A, a Venn diagram was used to show the intersections and distinct bacterial compositions among different experimental groups. To evaluate microbial diversity, we calculated the α‐diversity index, specifically the Chao1 index, which serves as an indicator of bacterial richness. Our findings revealed a significant increase in the Chao1 index in MPTP‐induced PD mice (Figure 8B), consistent with previous studies indicating that higher α‐diversity is often associated with gut microbiota alterations in PD [57, 58, 59]. Notably, treatment with the *L. plantarum*@MPN@CS formulation resulted in a significant reduction in the Chao1 index, restoring it to levels comparable to those observed in healthy control mice. This suggests that *L. plantarum*@MPN@CS effectively reshapes the gut microbiota composition, changing it closer to a healthy, balanced state.

To further investigate the effects on microbial community structure, we performed nonmetric multidimensional scaling (NMDS) analysis based on a Bray‐Curtis distance matrix to assess the similarity of gut microbiota compositions. The results revealed that the *L. plantarum*@MPN@CS‐treated group formed a distinct cluster compared to the PD model group, indicating a significant disruption in the gut microbiota of PD mice that could be effectively restored with oral administration of *L. plantarum*@MPN@CS (Figure 8C). To gain deeper insights, we analyzed the differences in gut microbiota composition across experimental groups at the phylum level. We observed a significant increase in the relative abundance of Firmicutes in the PD model group, as reported previously [60], leading to an elevated Firmicutes/Bacteroidetes (F/B) ratio (Figure 8D). However, the dysbiosis was improved following *L. plantarum*@MPN@CS supplementation. At the family level, *Bifidobacterium*, a well‐established probiotic known for producing lactic acid to maintain a low‐pH environment in the GI tract and limit pathogen growth [61], was significantly enriched in the *L. plantarum*@MPN@CS‐treated group (Figure 8E). To further assess the taxonomic impact of the treatment, we performed linear discriminant analysis effect size (LEfSe) analysis (Figure 8F,G). This analysis revealed significant differences in the gut microbiota composition between the PD model group and the *L. plantarum*@MPN@CS‐treated group. Specifically, *Bacteroides* and Tannerellaceae were more abundant in PD mice, while these potentially harmful taxa were markedly reduced following *L. plantarum*@MPN@CS treatment. Conversely, beneficial probiotics such as *Lactobacillus* and *Bifidobacterium* were significantly enriched in the treated group. Collectively, these results demonstrate that *L. plantarum*@MPN@CS can effectively increase the relative abundance of beneficial bacteria, while simultaneously reducing harmful bacteria, thereby restoring gut microbiota balance and alleviating PD symptoms.

### 2.9 Biosafety Evaluation of *L. plantarum*@MPN@CS

The biosafety of *L. plantarum*@MPN@CS was rigorously evaluated to assess its potential for clinical application. Thirty days post‐administration, mice treated with *L. plantarum*@MPN@CS were sacrificed, and histological analysis was performed on major organs, including the heart, liver, spleen, lungs, and kidneys. As anticipated, no significant signs of inflammatory response were observed in the treated group compared to the untreated control group (Figure S20).

Furthermore, routine blood analysis showed no abnormalities in liver and kidney function markers (Figure S21), further underscoring the high safety profile of *L. plantarum*@MPN@CS. Collectively, these results demonstrate that the probiotic, encapsulated in a microenvironment self‐adaptive nanoarmor, possesses excellent safety characteristics, making it a promising candidate for therapeutic interventions in PD.

## 3 Conclusion

In conclusion, we successfully developed a microenvironment self‐adaptive nanoarmor on *L. plantarum*, creating a highly functional super probiotic system (*L. plantarum*@MPN@CS) through a simple and adaptable assembly method. This system exhibits exceptional resistance to gastrointestinal stresses, ensuring its robust functionality in dynamic and challenging environments. The incorporation of partially acetylated CS endowed the nanoarmor with unique self‐adaptive properties, which effectively addressed adhesion and colonization challenges within complex physiological and pathological microenvironments. By integrating the intestinal microenvironment self‐adaptive nanoarmor with the inherent anti‐inflammatory properties of *L. plantarum*, the super probiotic system was able to alleviate intestinal inflammation, restore gut barrier integrity, and reestablish microbial homeostasis. Consequently, the nanocoated probiotic demonstrated remarkable therapeutic potential in the treatment of PD, as evidenced by its ability to improve motor behavior disorders, reduce dopaminergic neuronal death, and mitigate neuroimmune responses in a mouse model. These findings collectively highlight the importance of strategies that manipulate bacterial colonization to modulate host‐microbiota interactions, particularly in improving extraintestinal disorders.

The success of this study highlights the significant potential of a microenvironment self‐adaptive nanoarmor‐assisted probiotic delivery system in developing bacterial therapeutics for extraintestinal diseases, as exemplified by its application in PD management. This innovative approach addresses the critical challenges related to bacterial adhesion and colonization in impaired intestinal environments, offering a promising strategy to enhance probiotic efficacy in complex pathological conditions. Notably, the use of inflammation‐related secreted glycoproteins, such as CHI3L1, as colonization niches represents a novel and effective strategy to mitigate competitive inhibition and reduce potential side effects associated with increased epithelial‐adherent microbial communities in gut inflammatory microenvironments. This strategy ensures improved bacterial colonization and proliferation under dynamic and hostile intestinal conditions, providing a robust platform for advancing targeted therapies for extraintestinal diseases.

Despite these promising results, several limitations remain to be addressed in future studies. First, while this study focused on enhancing probiotic efficacy in PD, the broader applicability of this strategy to other extraintestinal disorders requires further exploration. Second, additional validation of the microenvironment self‐adaptive nanoarmor‐assisted probiotic delivery system in diverse animal models is crucial to thoroughly confirm its efficacy and safety. Lastly, a deeper understanding of the underlying molecular mechanisms, particularly the pathways through which *L. plantarum*@MPN@CS alleviates PD symptoms, is essential for optimizing therapeutic outcomes. Future research should aim to expand the system's applicability, enhance its adaptability, and elucidate the molecular basis of its therapeutic effects, ultimately paving the way for the development of more effective and versatile living therapeutic strategies for extraintestinal diseases.

## 4 Experimental Section

### 4.1 Materials

The chemicals and biological reagents used in this investigation are as follows: procyanidin (PC, Macklin), iron (III) chloride (FeCl_3_, Aladdin), 1‐methyl‐4‐phenyl‐1,2,3,6‐tetrahydropyridine hydrochloride (MPTP, Aladdin), polyethyleneimine (PEI, M.W. 1800, Aladdin), LIVE/DEAD BacLight Bacterial Viability Kits (Invitrogen), Rhodamine B (Sigma–Aldrich), transferrin (Sigma–Aldrich), recombinant mouse CHI3L1 protein (Sino Biological Inc.), anti‐fade mounting medium containing 4′,6‐diamidino‐2‐phenylindole (DAPI, Life Technologies, Waltham, MA, USA), bile salts (Sigma–Aldrich), porcine pancreatic trypsin (Sigma–Aldrich), pepsin powder (Aladdin), MRS medium (Hopebio), and primary antibodies (anti‐CHI3L1, anti‐ZO1, anti‐occludin, anti‐TH, anti‐Iba‐1, all from Abcam). Secondary antibodies included goat anti‐rabbit IgG H&L (Alexa Fluor 488), goat anti‐mouse IgG H&L (Alexa Fluor 488), and donkey anti‐rabbit IgG H&L (Alexa Fluor 647, Abcam). Enzyme‐linked immunosorbent assay (ELISA) kits for mouse tumor necrosis factor‐α (TNF‐α), interleukin‐1β (IL‐1β), and interleukin‐6 (IL‐6) were obtained from Solarbio. Partially acetylated Chitosan oligosaccharides (CS) were prepared as previously reported, with a deacetylation degree of 44%. Unless otherwise specified, all additional reagents were of the highest purity available from local suppliers and were used as received without further refinement.

### 4.2 Bacterial Strains and Animals

Male C57BL/6J mice (6 weeks old, weighing 20 ± 2 g) were purchased from the Experimental Animal Center of Zhejiang Province (Zhejiang, China). The mice were acclimatized for 7 days in a controlled environment (temperature and humidity) with a 12 h light/dark cycle, and had ad libitum access to sterilized food and water. All experimental procedures were conducted in compliance with the China Public Health Service Guide for the Care and Use of Laboratory Animals and were approved by the Institutional Animal Care and Use Committee of the Wenzhou Institute, University of Chinese Academy of Sciences (approval number: WIUCAS23041701). Throughout the study, the mice were housed in a specific‐pathogen‐free (SPF) environment.

The probiotic strain *Lactobacillus plantarum* ST‐III AB161 (*L. plantarum*) was obtained from the China General Microbiological Culture Collection Center (CGMCC 22 782) and provided by the Biological Experimental Center of Wenzhou Medical University.

### 4.3 Preparation of Coated Probiotics

*L. plantarum* was cultured in 50 mL of MRS medium at 37°C for 12 h. The bacterial solution (2 × 10^8 CFU/mL) was centrifuged and washed three times with 0.15 m NaCl, then resuspended in 1 mL of the same saline solution. Procyanidine (PC, 1 mg/mL) and FeCl_3_ (0.5 mg/mL) were dissolved in Tris‐HCl buffer (0.01 m, pH 8.2). 1 mL of FeCl_3_ solution and 3 mL of PC solution were slowly added to the *L. plantarum* solution drop by drop and incubated at 37°C with shaking at 150 rpm for 0.5 h, yielding *L. plantarum*@MPN. To fluorescently lable the MPN layers, *L. plantarum*@MPN was incubated with Rhodamine B‐BSA (0.2 mg/mL) for an additional 30 min. The resulting *L. plantarum*@MPN was centrifuged, washed three times with 0.15 m NaCl to remove free PC and FeCl_3_, and resuspended in 5 mL of partially acetylated CS solution (5 mg/mL). The suspension was stirred at 37°C for 1 h to allow sequential CS layer deposition, resulting in the final *L. plantarum*@MPN@CS construct using a layer‐by‐layer coating approach. Alternatively, polyethylenimine (PEI) was used instead of partially acetylated CS to prepare *L. plantarum*@MPN@PEI by the same method.

### 4.4 Characterization of Coated Probiotics

The zeta potential and size distribution of native *L. plantarum*, *L. plantarum*@MPN, and *L. plantarum*@MPN@CS were measured using a Zetasizer Nano ZS instrument (Malvern ZEN3600, UK). Morphological analysis was performed using transmission electron microscopy (TEM, FEI Talos F200S, UK). For TEM, bacterial solutions were placed on glow‐discharged, carbon‐coated copper grids (200 mesh) for 5 min, followed by air‐drying. The MPN layer was tagged with Rhodamine B‐labeled bovine serum albumin (BSA), while the partially acetylated CS layer was visualized using FITC‐conjugated CS. Confocal laser scanning microscopy (CLSM, Nikon Eclipse TE 200‐S, Japan) was employed to further characterize the encapsulation of *L. plantarum*.

### 4.5 Measurement of Growth Curves of Coated Probiotics

To evaluate the effect of surface nanocoating on bacterial growth, growth curves were measured for native *L. plantarum*, *L. plantarum*@MPN, and *L. plantarum*@MPN@CS. Each probiotic formulation was diluted in 10 mL of fresh MRS medium to an initial optical density at 600 nm (OD_600_) of 0.2. The cultures were incubated at 37°C with 5% CO_2_, and OD_600_ was recorded at hourly intervals over 20 h using a microplate reader (Varioskan LUX, USA).

### 4.6 Evaluation of Resistance to Simulated Gastric and Intestinal Conditions

To assess resistance to simulated gastric and intestinal conditions, equivalent native *L. plantarum*, *L. plantarum*@MPN, and *L. plantarum*@MPN@CS (5 × 10^7 CFU) were resuspended in simulated gastric fluid (SGF, pH 2.0, with pepsin), simulated intestinal fluid (SIF, pH 6.8, with trypsin), or bile acid (0.3 mg/mL) and incubated at 37°C with gentle shaking. At predetermined time points, 50 µL samples were collected, washed with 0.15 m NaCl, diluted, and plated on MRS agar. Colonies were counted after 24 h of incubation at 37°C. Bacterial viability was further assessed using LIVE/DEAD BacLight Kits according to the manufacturer's instructions. Additionally, the morphological changes of naked or coated *L. plantarum* following SGF treatment were observed via TEM.

### 4.7 Radical Scavenging Study In Vitro

The DPPH radical scavenging activity was assessed using the 2,2‐Diphenyl‐1‐picrylhydrazyl (DPPH) probe. DPPH was dissolved in methanol, and native *L. plantarum*, *L. plantarum*@MPN, and *L. plantarum*@MPN@CS were incubated with 1 mL of DPPH solution (0.15 mg/mL) in the dark at 37°C with gentle shaking for 10 min. The remaining DPPH concentration was determined by measuring UV absorbance at 517 nm. To evaluate ABTS radical scavenging activity, an ABTS free radical scavenging assay kit (Solarbio Science & Technology) was used according to the manufacturer's instructions. The capacity of native *L. plantarum*, *L. plantarum*@MPN, and *L. plantarum*@MPN@CS to eliminate ABTS radicals was quantified by detecting changes in UV absorbance at 405 nm.

### 4.8 Ex Vivo Adhesion Studies

Freshly collected intestinal tissues from PD mice were cut into ∼1 cm segments and everted to expose the intestinal lumen and mucosal layer. Equal amounts of native *L. plantarum*‐mCherry (carrying pMG36e‐Px‐mCherry, erythromycin resistance), *L. plantarum*‐mCherry@MPN, and *L. plantarum*‐mCherry@MPN@CS (5 × 10^6 CFU) were applied to the surface of the intestinal segments. After 30 min of incubation at 37°C with gentle rotation (30 rpm), the segments were washed gently with PBS to remove any unattached bacteria. The segments were then imaged using an IVIS system (IVIS Lumina XRMS Series III, USA).

### 4.9 In Vivo Intestinal Retention Experiment

To evaluate bacterial retention in the gastrointestinal tract, mice were orally administered *L. plantarum*‐mCherry, *L. plantarum*‐mCherry@MPN, and *L. plantarum*‐mCherry@MPN@CS (1 × 10^8 CFU) via gavage. Fluorescent images were captured at various time points (0.5, 3, 12, 24, 48, 72, and 96 h) post‐administration. For ex vivo imaging, mice were euthanized under deep anesthesia, and the intestines were immediately harvested for imaging. The total radiant efficiency of the fluorescence signal was quantified using Living Image Analysis Software (IVIS Lumina XRMS Series III, USA). Fluorescence data were further analyzed using the specific longitudinal data analysis tools (Region of Interest‐ROI tool) to enable efficient and accurate quantification of the calibrated fluorescence signals. Additionally, intestinal tissues and contents were collected, homogenized in 1 mL PBS, serially diluted, and plated on MRS agar to determine colony‐forming units (CFU).

### 4.10 Binding Affinity Measurement via Microscale Thermophoresis

Proteins CHI3L1 and transferrin were labeled with the Monolith RED‐NHS Protein Labeling Kit (NanoTemper Technologies, Munich, Germany) following the manufacturer's protocol. The labeled proteins were used at a final concentration of 4 µm, while the non‐fluorescently labeled partially acetylated CS ligands were titrated in a 1:1 dilution series, spanning concentrations from 400 µM to 12.2 nm. The samples were subsequently loaded into Monolith NT.115 MST standard‐treated capillaries (NanoTemper Technologies) and immediately subjected to measurement by Microscale Thermophoresis (MST). Temperature was maintained at 37°C by the control software following proper mixing of the samples. Experimental parameters for MST were set to 20% LED power and 40% MST power. Binding affinity data were derived from triplicate measurements of independently prepared samples. The binding affinity (*K_D_* value) was calculated using the MO‐Affinity Analysis software (version 2.1.3, NanoTemper Technologies).

### 4.11 MPTP‐Induced Model of PD

The subacute PD model was established by intraperitoneal administration of MPTP, as previously described with slight modifications [62], Briefly, male C57BL/6J mice (5–6 weeks old) were subjected to a daily intraperitoneal injection of MPTP (30 mg/kg, dissolved in saline) for 7 consecutive days, with a 24 h interval between each injection. This treatment regimen was designed to reliably induce PD‐like symptoms. For the control group, an equivalent volume of saline was administered.

### 4.12 Behavioral Assessments

The behavior tests were assessed according to a previously reported user's guide with some modifications [44]. Behavioral studies were conducted in a blinded manner between 8:00 AM and 10:00 PM, with all tests performed under light conditions. Mice were acclimatized to the behavioral testing room for at least 1 h prior to testing. All behavioral apparatuses were thoroughly cleaned and wiped with 75% ethanol to eliminate any odor residues left by the previous animal.

#### 4.12.1 Rotarod Test

Motor coordination and balance were evaluated using a Rotarod apparatus with separate chambers. The first 3 days were designated as training days, while the fourth day was the test day. Mice were initially placed on a 3 cm‐diameter rotating cylinder and allowed to adapt by remaining on the stationary rod for 5 min during the trial sessions. Following this, the mice were gradually acclimated to the rotating rod by setting it to rotate slowly at 5 revolutions per minute (RPM) without acceleration. If the animal fell off, it was immediately placed back on the rod, with a maximum of five attempts per session. After 3 days of training on the slowly rotating rod, the experimental procedure began. During the test, a constant acceleration mode was employed. Each mouse was placed on the rod, which started rotating at 5 RPM and gradually accelerated to 40 RPM over a 5 min period. The duration of mice staying on the rod was recorded, and the test was repeated three times at 1 h intervals.

#### 4.12.2 Gait Analysis

Gait characteristics were assessed using a footprint method. During the training phase, mice were placed at one end of a narrow corridor, with a dark box positioned at the opposite end. Given their natural preference for dark environments, the mice were allowed to walk from the start of the corridor to the dark box, completing one trial. Training sessions were conducted three times daily, with 15 min intervals between sessions, over the course of three consecutive days. In the experimental phase, the fore and hind paws of the mice were painted with red and blue dyes, respectively. The mice were then encouraged to walk in a straight line along the narrow corridor, which was lined with absorbent paper, toward the dark box. Upon reaching the dark box, the paper was removed and allowed to dry. This procedure was repeated for all animals in the study. Footprint patterns were subsequently analyzed to measure various gait parameters, including stride length, base width, overlap between fore and hind paws, and paw splay. At least four footprints from each mouse were required for data analysis. Typically, the first few footprints were excluded from statistical analysis, as the mice may not yet be in a continuous walking phase at this point.

#### 4.12.3 Open Field Test

Locomotor activity and exploratory behavior were evaluated in an open field arena (40 cm × 40 cm × 30 cm). Mice were initially acclimatized to the arena for 10 min during the trial sessions. Training was conducted three times daily, with 30 min intervals between sessions. In the experimental phase, mice were randomly placed into the arena and allowed to explore freely for 5 min. Spontaneous exploratory activity was recorded using a TopScan automated video system. After each trial, the arena was sanitized with a 75% ethanol solution to eliminate scent cues left by the previous animal. Each mouse underwent three independent trials to assess locomotor activity and exploratory behavior.

### 4.13 Biosafety Evaluation

To evaluate the safety of orally administered *L. plantarum* @MPN@CS, mice were gavaged daily with 200 µL of the bacterial suspension (5 × 10^8 CFU) for 30 days. Native *L. plantarum* was used as a control. Following euthanasia, tissue samples (heart, liver, spleen, lungs, and kidneys) were collected, fixed in 4% paraformaldehyde, and stained with hematoxylin and eosin (H&E) for histological analysis. Concurrently, blood samples were collected to assess liver and kidney function, quantifying markers including alanine aminotransferase (ALT), aspartate aminotransferase (AST), creatinine (CR), and blood urea nitrogen (BUN) levels. These analyses ensured a comprehensive safety profile, adhering to rigorous scientific standards.

### 4.14 Prophylactic Efficacy of *L. plantarum*@MPN@CS Against MPTP‐Induced PD Syndromes

Male C57BL/6J mice (*n* = 5 per group) were randomly assigned to five experimental groups: PBS, PD, PD + *L. plantarum*, PD + *L. plantarum*@MPN, and PD + *L. plantarum*@MPN@CS. The experimental protocol involved an initial 7 day period of oral administration of PBS or bacterial suspensions containing naked or coated *L. plantarum* (5 × 10^8 CFU per dose). Meanwhile, all groups, except the PBS control, underwent a subacute PD induction via intraperitoneal MPTP administration (30 mg/kg) for 7 consecutive days to induce PD‐like symptoms.

Throughout the experimental period, mice continued to receive their assigned treatments for an additional 7 days, corresponding to their group assignment. Specifically, the PBS group received PBS only, while the remaining groups were treated with the following: native *L. plantarum* (PD + *L. plantarum*), *L. plantarum* encapsulated in MPN (PD + *L. plantarum*@MPN), and *L. plantarum* encapsulated in both MPN and partially acetylated CS (PD + *L. plantarum*@MPN@CS). The treatments were administered daily by oral gavage, ensuring consistent dosing for all animals.

### 4.15 Intestinal Permeability Detection In Vivo

The in vivo intestinal permeability assays were performed using fluorescein isothiocyanate (FITC)‐dextran (4 kDa, Sigma) as previously described [53]. After a 4 h period of food and water deprivation, mice were orally gavaged with FITC‐dextran (0.6 g/kg body weight). Serum samples were then collected after 6 h and analyzed to determine the FITC‐dextran concentration.

### 4.16 Tissue Collection

Following completion of the behavioral assessments, all mice were euthanized under deep anesthesia using an overdose of isoflurane, in accordance with ethical guidelines. Blood was collected via enucleation of the eyes, and the specimens were immediately transferred to Eppendorf tubes. The blood samples (approximately 500 µL) were left at room‐temperature for 2 h to allow natural clotting. Afterward, serum was separated by centrifugation at 2500 g for 15 min at room‐temperature. The resulting supernatant (100 µL) was carefully harvested for subsequent analysis.

The brains were carefully excised and coronally sectioned into approximately 1 mm thick segments using Brain Matrices (Servicebio SQP‐H12), as illustrated in Figure S25. Given the well‐established effect of MPTP administration on the substantial loss of tyrosine hydroxylase (TH)‐positive neurons in both the substantia nigra (SN) and striatum as referenced in previous studies [63], regions 6 and 4 of the brain were selected for further analysis. Immunofluorescence staining analysis was performed to assess the expression levels of TH and Iba1‐positive microglial cells in the SN and striatum.

The intestine sections were fixed, embedded in paraffin, and processed for histopathological staining. And cecal contents were collected for gut microbiome profiling via 16S rDNA amplicon sequencing, and remaining brain and colon segments were stored at ‐80°C for future analysis.

### 4.17 Histopathology and Immunofluorescence Analysis

Tissues were fixed in 4% paraformaldehyde and subsequently embedded in paraffin. Intestinal samples were sectioned into 4 µm thick slides, deparaffinized, and processed for hematoxylin and eosin (H&E) staining following the manufacturer's instructions. The injury score was assessed according to previously established criteria, with a scoring system provided in the Table S1. This system included parameters such as the severity of inflammation, depth of injury, extent of crypt damage, and percentage of tissue involvement. To evaluate mucosal barrier damage in PD‐related pathological conditions, Alcian blue (AB) and Periodic Acid‐Schiff (PAS) staining were performed.

For immunofluorescence analysis, tissue sections were deparaffinized in xylene and rehydrated through an ascending ethanol series. Antigen retrieval was carried out using citrate buffer (0.01 m, pH 6.0, 0.05% Tween‐20) in a steamer at 95°C for 20 min. Endogenous peroxidase activity was blocked by treating the sections with 3% hydrogen peroxide in methanol for 10 min. After three washes with TBS, the sections were permeabilized and blocked with 10% normal goat serum in 0.3% Triton X‐100 PBST (PBS with 0.05% Tween‐20) for 1 h at room‐temperature. Brain sections were incubated overnight at 4°C with primary antibodies against tyrosine TH (1:50) and Iba1 (1:100), followed by incubation with the appropriate secondary antibodies for 1 h at room‐temperature. For intestinal sections, primary antibodies against occludin (1:200) and ZO‐1 (1:100) were applied using the same protocol. All sections were mounted with Antifade Mountant containing DAPI for nuclear staining. Finally, images were captured using CLSM (Nikon, Japan), and the stained areas in both brain and intestinal tissues were quantitatively analyzed using ImageJ software (National Institutes of Health, USA).

### 4.18 Real‐Time Quantitative PCR (RT‐qPCR)

Brain tissue samples were selected for total RNA extraction using TRIzon reagent. RNA concentration and purity were assessed on a Nanodrop 2000 spectrophotometer (Thermo Fisher Scientific, Waltham, MA, USA). A total of 1 µg RNA was reverse transcribed into cDNA using the HiFiScript cDNA Synthesis Kit (Beijing Cowin Biotech Co., Ltd., Beijing, China), following the manufacturer's instructions. RT‐qPCR was performed using the UltraSYBR Mixture Kit (Beijing Cowin Biotech Co., Ltd., Beijing, China). The specificity of the RT‐qPCR assay was confirmed by melting curve analysis. The relative expression of the target gene was calculated using the 2^−ΔΔCt^ method, with normalization to the housekeeping gene β‐actin. A list of primer sequences can be found in Table S2.

### 4.19 Measurements for Inflammatory Cytokines in the Serum

Serum levels of TNF‐α, IL‐6, and IL‐1β were measured using ELISA kits according to the manufacturer's protocols.

### 4.20 Gut Microbiota Profiling

Fecal microbial DNA was extracted using a Magnetic Soil and Stool DNA Kit. The V3‐V4 hypervariable region of 16S rDNA was amplified with barcoded primers and sequenced on the Illumina NovaSeq 6000 platform. Raw sequencing data were processed using Qiime2 (Version 2020.06), where sequencing and PCR errors were minimized, and denoising was performed using DADA2. This process included filtering, dereplication, chimera removal, and merging paired‐end reads to generate amplicon sequence variants (ASVs) for taxonomic profiling. ASV taxonomic classification was carried out using a Naive Bayes classifier trained on the Silva Database. Alpha diversity was assessed using the Chao1 index, while beta diversity was evaluated through nonmetric multidimensional scaling (NMDS) based on Bray‐Curtis dissimilarity.

### 4.21 Statistical Analysis

Data are expressed as means ± standard deviation (SD) and are representative of at least three independent experiments. Prior to statistical analysis, data were assessed for normality using the Shapiro–Wilk test and for homogeneity of variance using the F‐test. Following the verification of these assumptions, statistical significance was determined by an unpaired, two‐tailed Student's *t*‐test. Analyses were performed with GraphPad Prism 10.3.1. Significance was set at *p* < 0.05, with ^*^ denoting *p* < 0.05, ^**^ *p* < 0.01, ^***^ *p* < 0.001, and ^****^ *p* < 0.0001.

## Author Contributions

L.M.Z., Y.Y.W., and Y.J.C. contributed equally to this work. L.Z. contributed to conceptualization, data curation, funding acquisition, investigation, methodology, and writing – original draft. Y.W. contributed to data curation, investigation, methodology, and software. Y.C. contributed to data curation, methodology, and software. X.C. contributed to methodology and software. X.Z. contributed to conceptualization, funding acquisition, validation, and writing – review and editing. Y.L. contributed to supervision and validation. W.G. contributed to supervision, validation, and writing – review and editing.

## Conflicts of Interest

The authors declare no conflict of interest.

## Supporting information




**Supporting File**: advs73709‐sup‐0001‐SuppMat.docx.

## Data Availability

The data that support the findings of this study are available in the supplementary material of this article.
