# Supplementary material for: Microenvironment Self‐Adaptive Nanoarmor to Address Adhesion‐ and Colonization‐Related Obstacles in Impaired Intestine Promote Bacteriotherapy Against Parkinson's Disease
Source: Adv Sci (Weinh). 2026 Jan 14;13(17):e10628. doi: 10.1002/advs.202510628 (PMC13042830; doi:10.1002/advs.202510628)
Supplement: Supplementary file 1 — Supporting File: advs73709‐sup‐0001‐SuppMat.docx. [file ADVS-13-e10628-s001.docx]

**Supporting Information**

Microenvironment Self-Adaptive Nanoarmor to Address Adhesion- and Colonization-Related Obstacles in Impaired Intestine Promote Bacteriotherapy Against Parkinson’s Disease

Limeng Zhu^†^, Yuyu Wu^†^, Yingjie Chen^†^, Xiyi Chen, Xingjie Zan*, Yanlong Liu*, and Wujun Geng*

**L. Zhu, X. Zan**

Wenzhou Key Laboratory of Perioperative Medicine, Wenzhou Institute, University of Chinese Academy of Sciences, Wenzhou 325001, Zhejiang, P.R. China

**W. Geng**

Department of Anesthesiology, Wenzhou Central Hospital Affiliated to Wenzhou Medical University, Wenzhou, Zhejiang, P.R. China.

**Y. Wu, X. Chen, Y. Liu**

School of Mental Health, Wenzhou Medical University, Wenzhou 325001, Zhejiang, P.R. China

**Y. Chen**

Cixi Biomedical Research Institute, Wenzhou Medical University, Wenzhou 325001, Zhejiang, P.R. China

**W. Geng**

Department of Pain, The First Affiliated Hospital of Wenzhou Medical University, Wenzhou 325000, Zhejiang, P.R. China.

**W. Geng**

Oujiang Laboratory (Zhejiang Lab for Regenerative Medicine, Vision and Brain Health), Wenzhou Medical University, Wenzhou 325001, Zhejiang, P.R. China

**Y. Wu**

School of Medicine, Zhejiang University, Hangzhou 310020, Zhejiang, P.R. China

^†^ These authors contribute equally to this work.

* Corresponding authors. Email: xjzan2000@hotmail.com (X. Zan); benjaminlyl@wmu.edu.cn (Y. Liu); gengwujun@126.com (W. Geng)


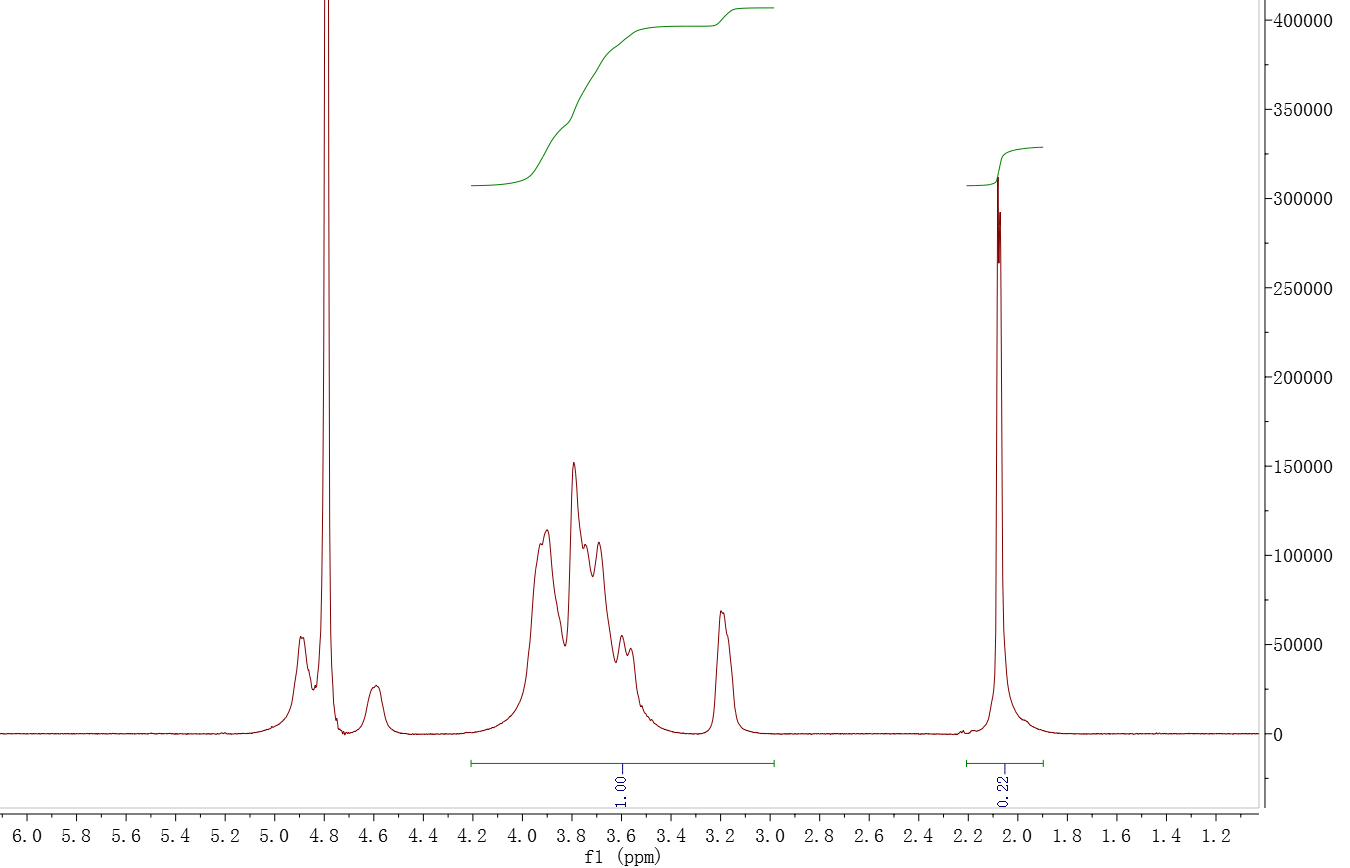


**Figure S1.** **^1^H NMR spectrum of partially acetylated CS.** The solutions of partially acetylated CS for NMR spectrum were prepared by stirring 10 mg of partially acetylated CS in a solution composed of 1 ml of D_2_O and waiting about 30 min at room temperature to ensure complete dissolution. ^1^H NMR spectra were acquired on an AVANCE III 600 MHz spectrometer (Bruker, Germany). The experiments were run at 70℃ at which the solvent (D_2_O) peak does not interfere with any of partially acetylated CS’s peaks. After dissolution, approximately 1 mL of the partially acetylated CS solution was transferred to a 5 mm NMR tube. The sample tube was inserted in the magnet and allowed to reach thermal equilibrium by waiting 10 min before experimenting.


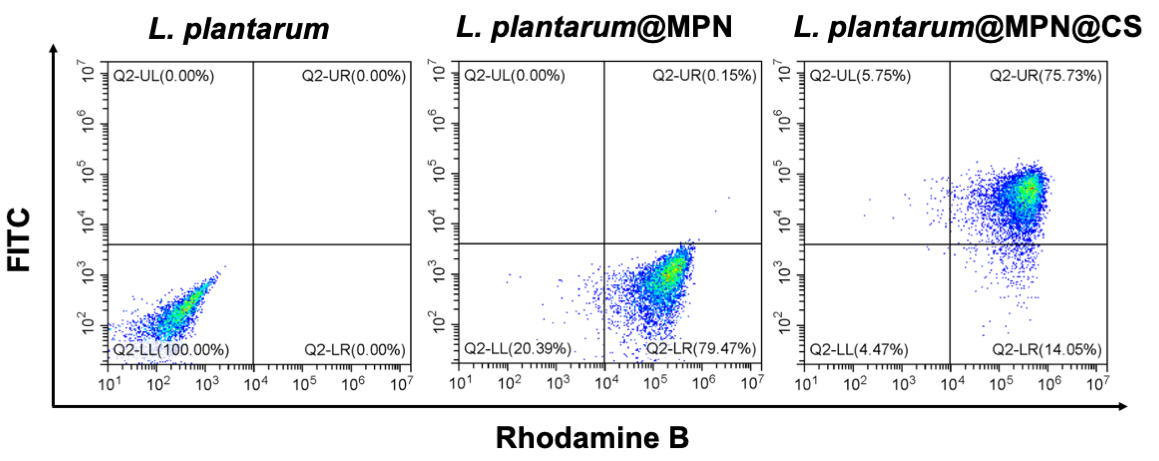


**Figure S2 Representative flow cytometry scatter plots and percentage of RhB^+^ and RhB^+^ FITC^+^ cells after coating.** Unlabeled *L. plantarum* served as controls. The red channel represents the Rhodamine B (RhB)-labeled MPN layer, and the green channel represents the FITC-labeled CS layer.

**
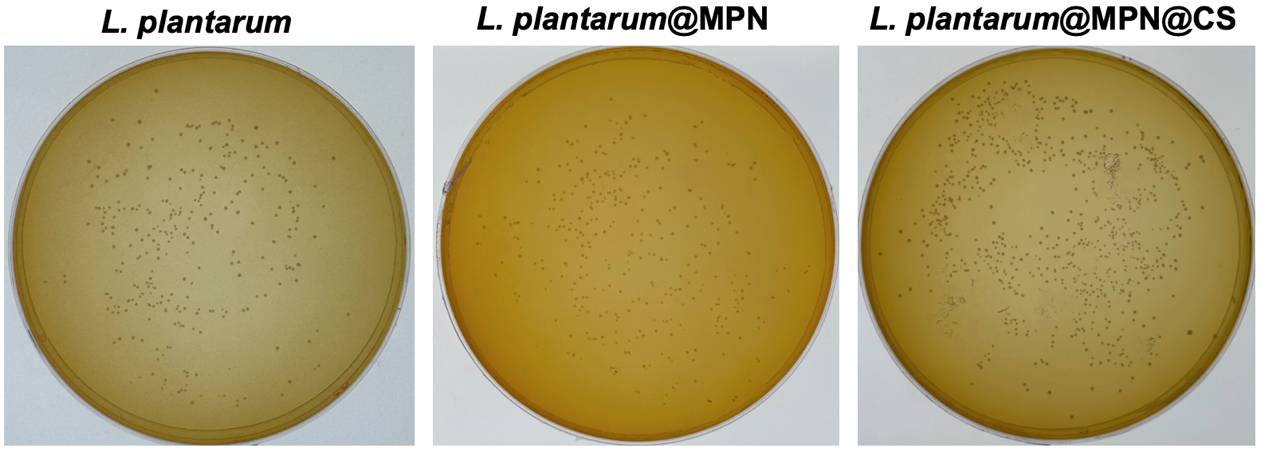
**

**Figure S3. Resistance of *L. plantarum*@MPN@CS to simulated intestinal fluid (SIF).** Representative photographs of bacterial colonies on agar plates for native *L. plantarum*, *L. plantarum*@MPN, and *L. plantarum*@MPN@CS after 2 h incubation in SIF (pH 6.8) supplemented with trypsin (10 mg/mL).

**
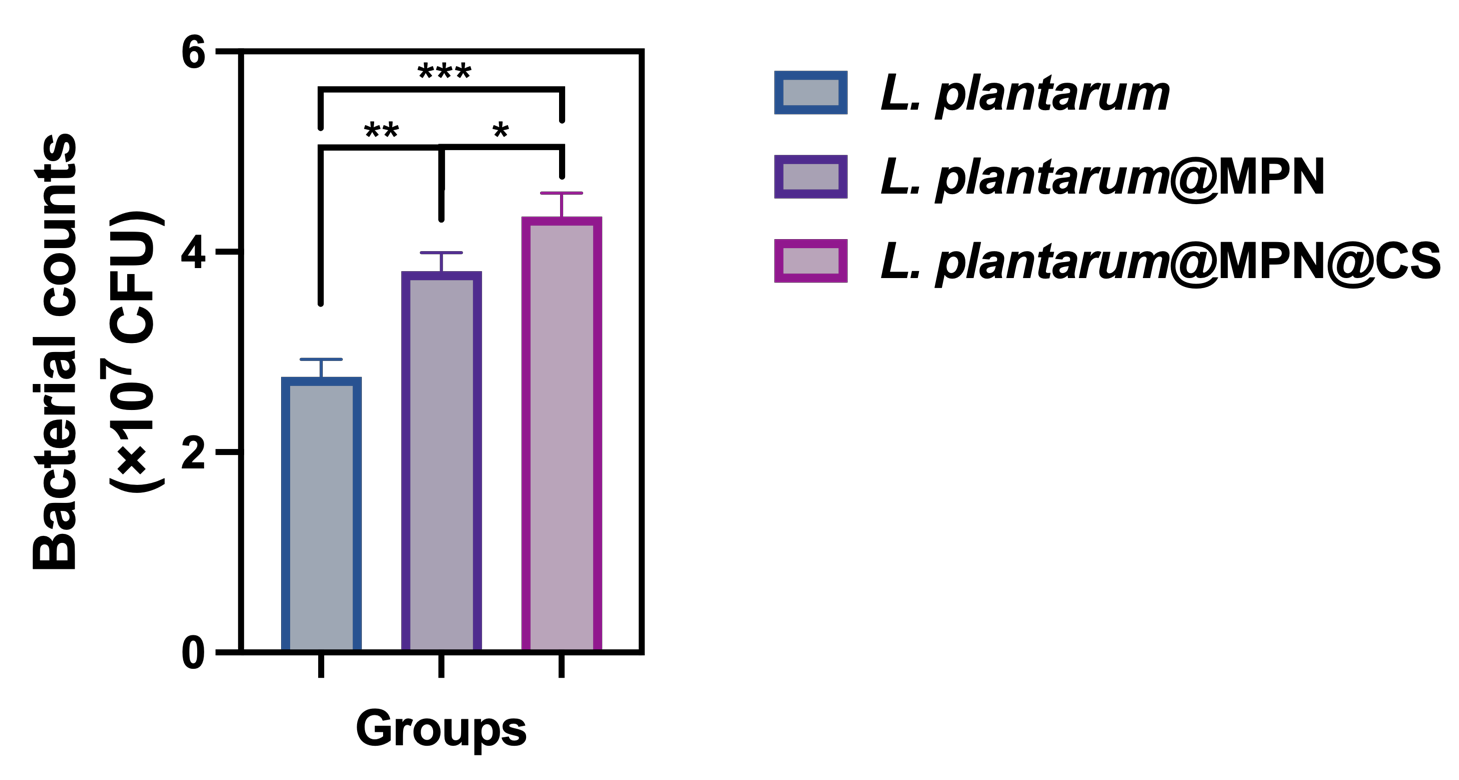
**

**Figure S4.** **Bacterial counts of naked and coated *L. plantarum* after exposure to SIF.** Data are presented as means ± SD (n=3 per group). Statistical significance was determined by an unpaired, two-tailed Student’s t-test. *P < 0.05, **P < 0.01, and ***P < 0.001.

**
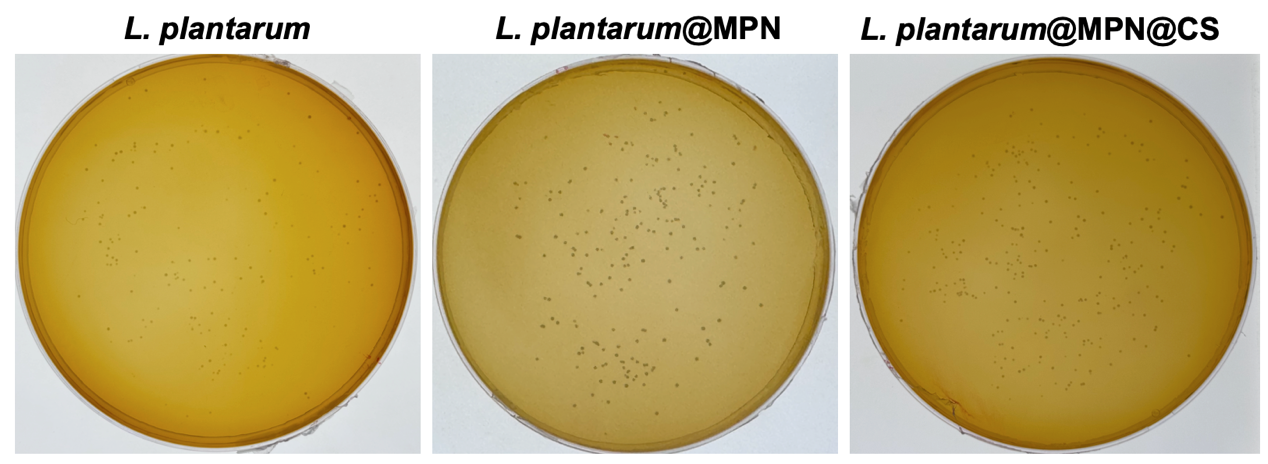
**

**Figure S5.** **Evaluation of *L. plantarum*@MPN@CS against bile acid*.*** Representative photographs of bacterial colonies formed on agar plates of native *L. plantarum*, *L. plantarum*@MPN, and *L. plantarum*@MPN@CS after 1 h incubation with bile acid (0.3 mg/mL).

**
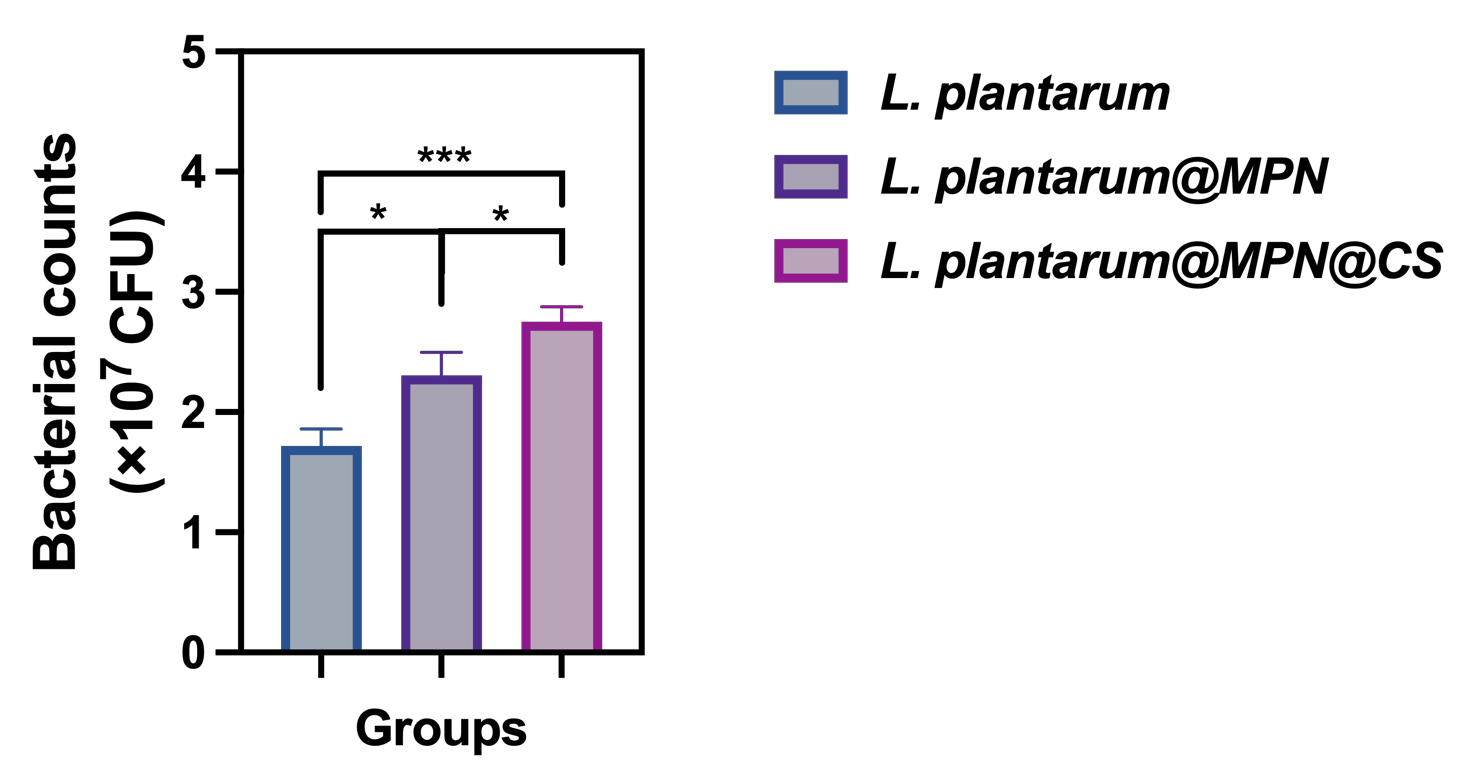
**

**Figure S6.** **Bacterial counts of naked and coated *L. plantarum* after exposure to bile acid.** Data are presented as means ± SD (n=3 per group). Statistical significance was determined by an unpaired, two-tailed Student’s t-test. *P < 0.05, ***P < 0.001.


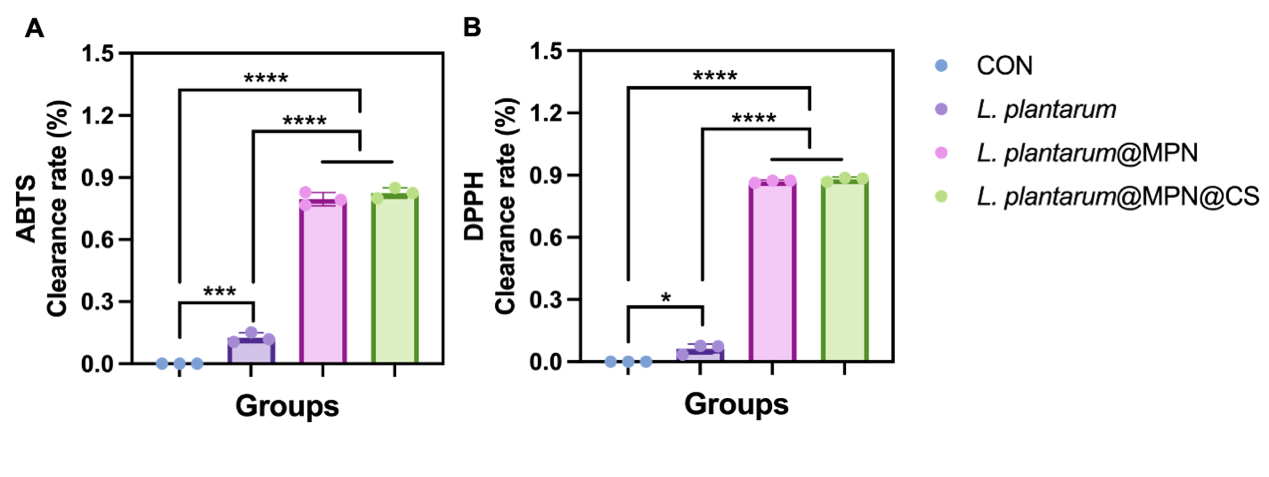


**Figure S7.** **The ABTS (A) and DPPH (B) scavenging properties of *L. plantarum* coated with nanoarmor.** Data are presented as means ± SD (n=3 per group). Statistical significance was determined by an unpaired, two-tailed Student’s t-test. *P < 0.05, ***P < 0.001, and ****P < 0.0001.


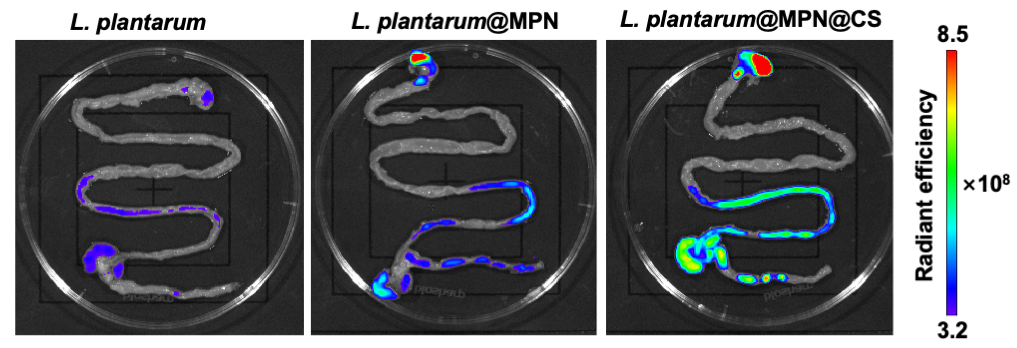


**Figure S8 Representative IVIS images of GI tracts 12 h post-administration.**


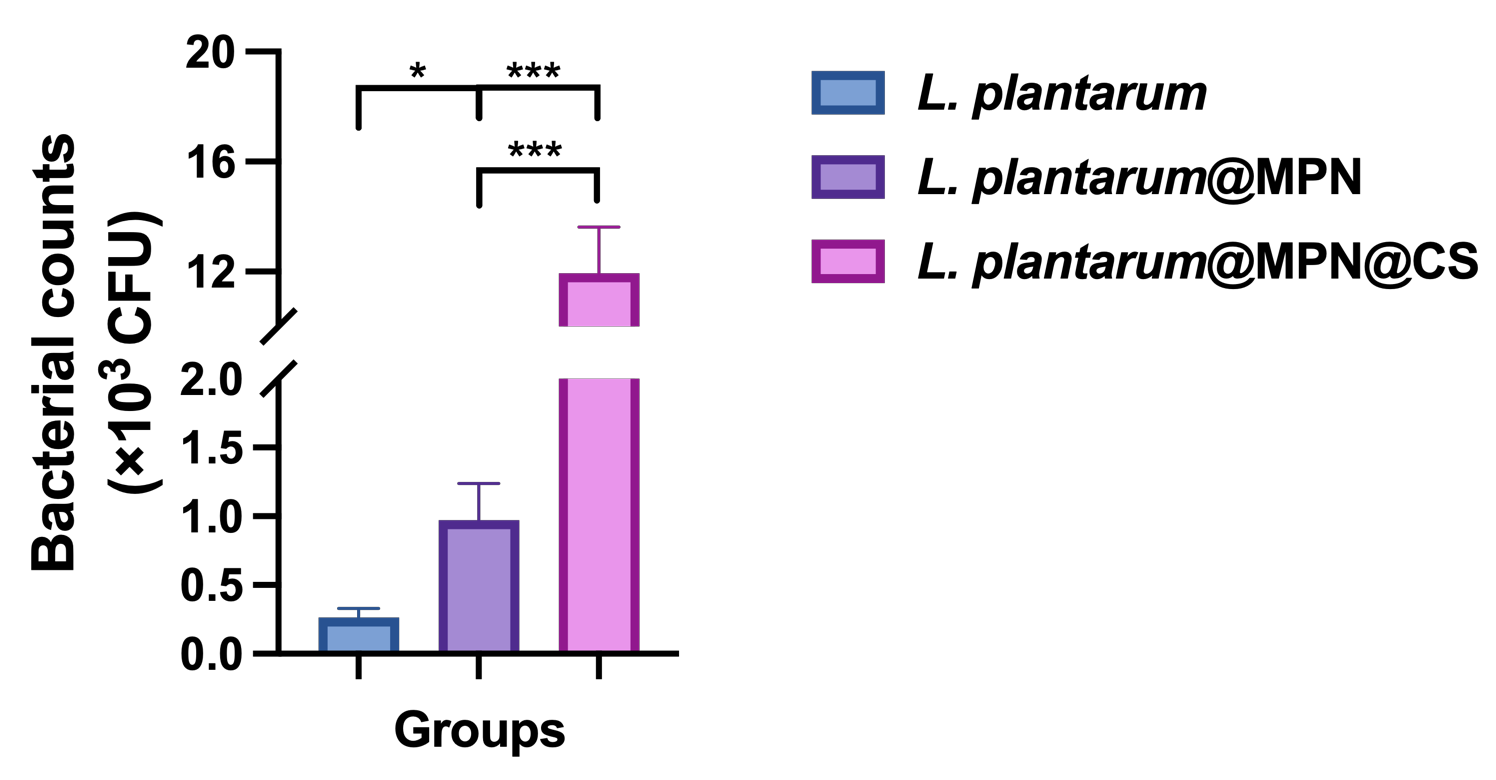


**Figure S9. Bacterial colonization of naked and coated *L. plantarum* in the ileum.** Data are presented as means ± SD (n = 3 per group). Statistical significance was determined by unpaired, two-tailed Student's t-test. *P < 0.05 and ***P < 0.001.


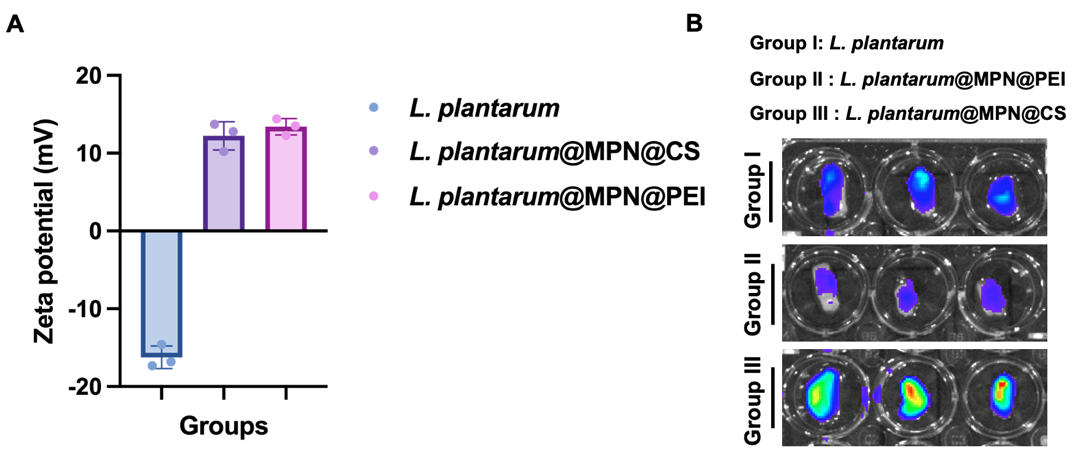


**Figure S10 Evaluation the relative contribution of electrostatic charge effects versus non-charge effects imparted by the partially acetylated CS modification on overall colonization efficiency under PD-related pathological conditions. (**A) Zeta potentials of naked *L. plantarum*, *L. plantarum*@MPN@PEI, and *L. plantarum*@MPN@CS measured by DLS. (B) Representative IVIS images of everted murine intestine segments incubated with naked or coated *L. plantarum* expressing mCherry. Data are presented as means ± SD (n = 3 per group).


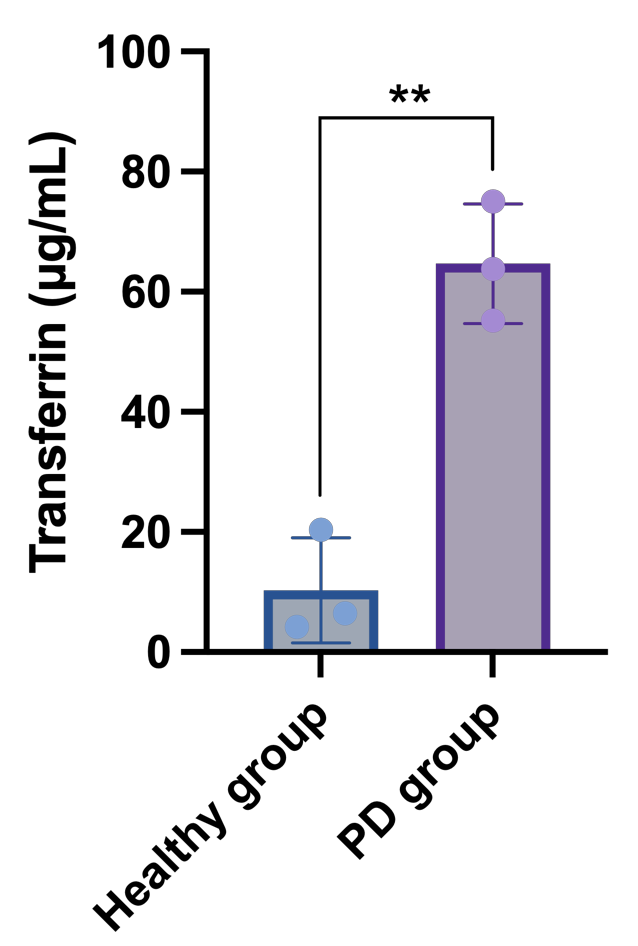


**Figure S11. The expression level of transferrin the PD gut milieu.** Data are presented as means ± SD (n=3 per group). Statistical significance was determined by an unpaired, two-tailed Student’s t-test. **P < 0.01.


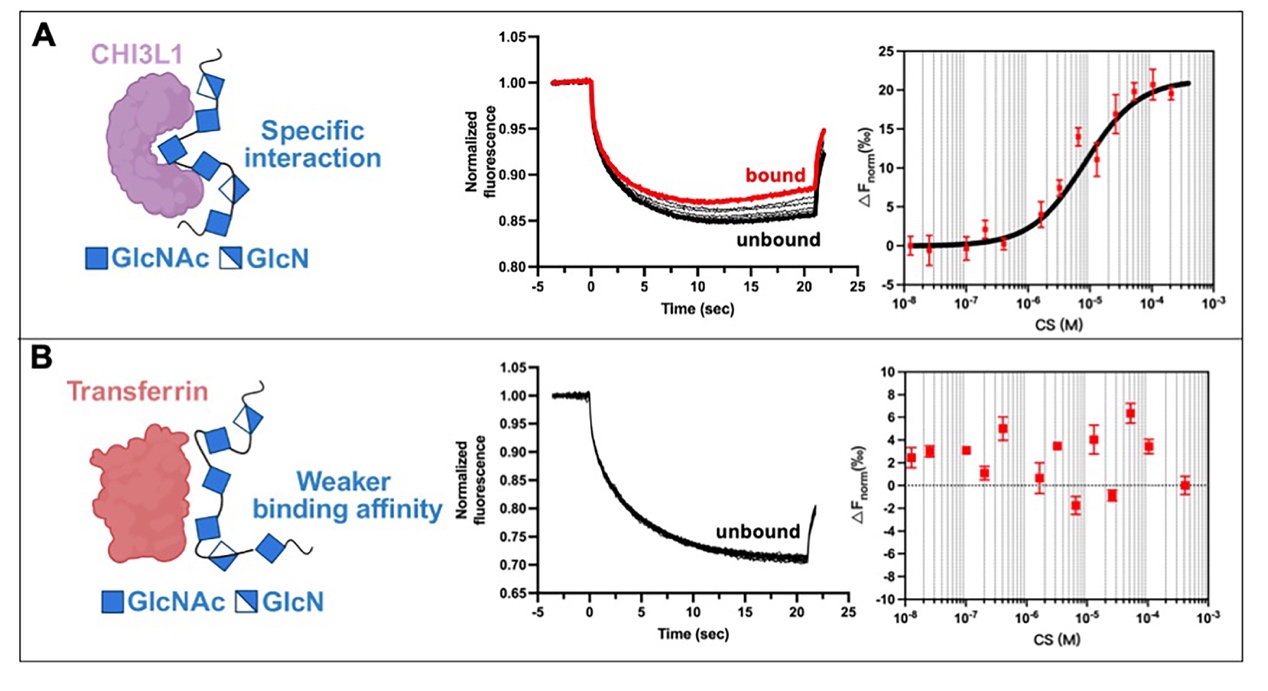


**Figure S12.** MST analysis of partially acetylated CS binding to (A) CHI3L1 and (B) transferrin.


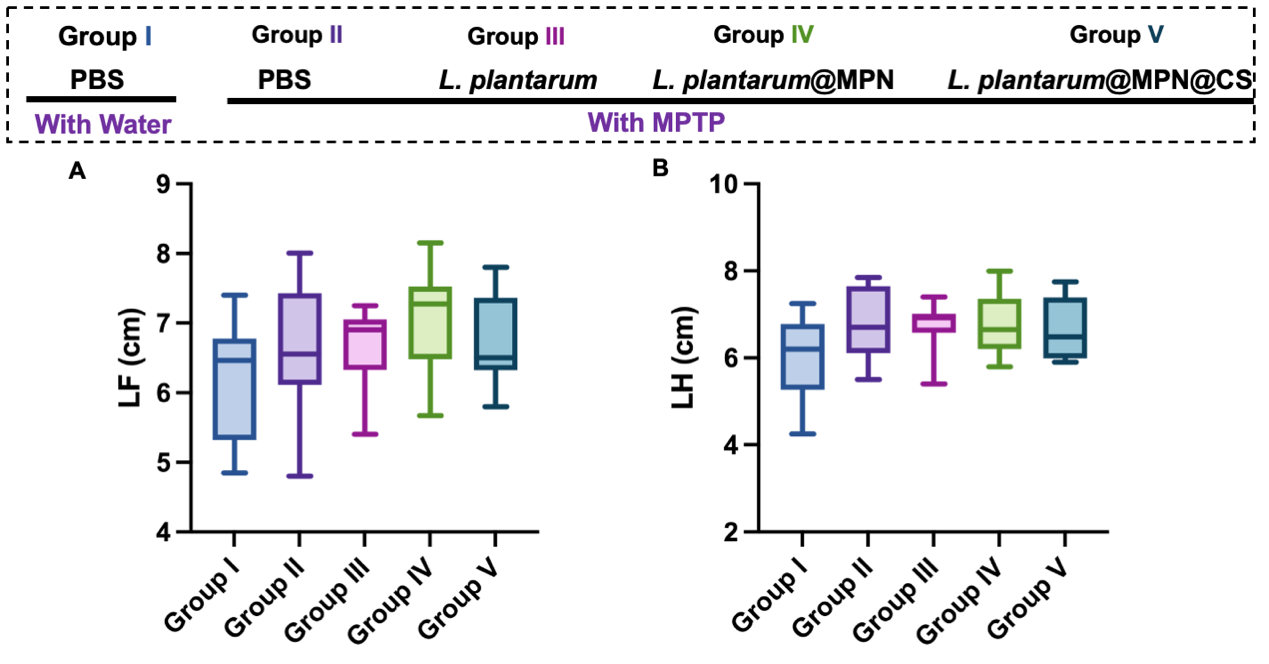


**Figure S13.** **Motor impairments assessed by gait analysis in MPTP-induced subacute PD model.** Stride lengths were measured for the left forelimb (LF) and left hindlimb (LH) in different group (n=5 per group).

**
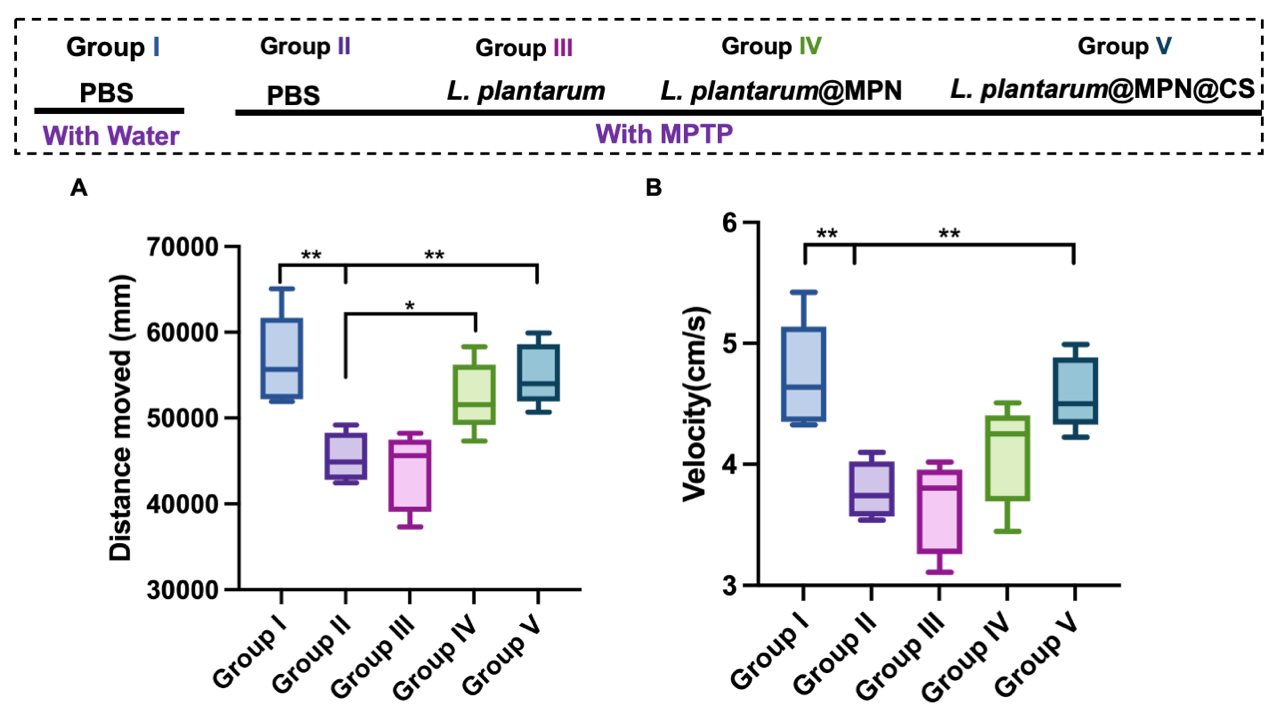
**

**Figure S14.** **Statistical analysis of the moving distance and velocity in the open field test.** Data are presented as means ± SD (n=5 per group). Statistical significance was determined by an unpaired, two-tailed Student’s t-test. *P < 0.05 and **P < 0.01.

**
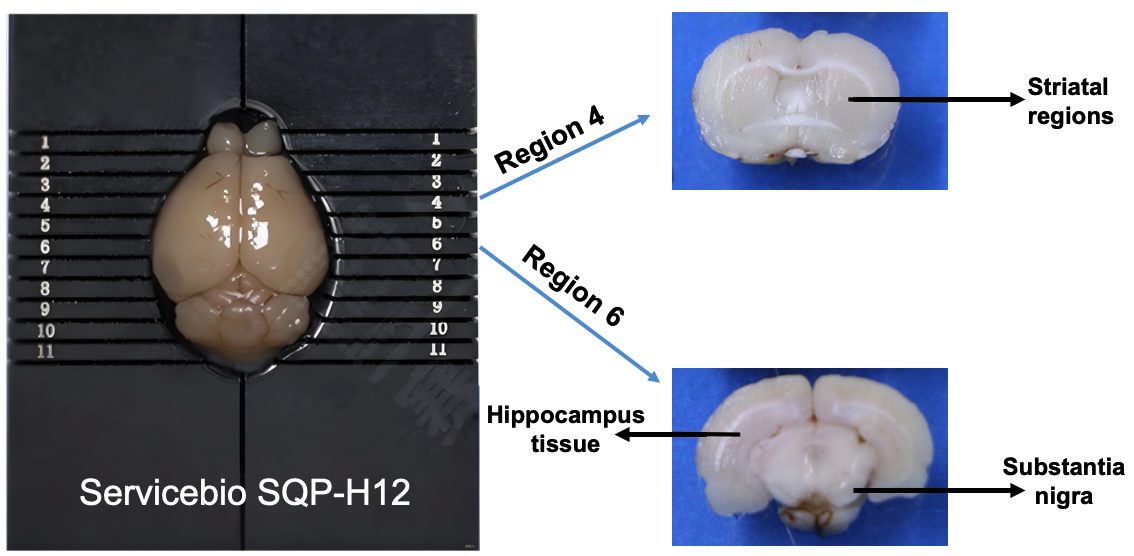
**

**Figure S15.** **Coronal sectioning of brain tissue using a brain matrix.**


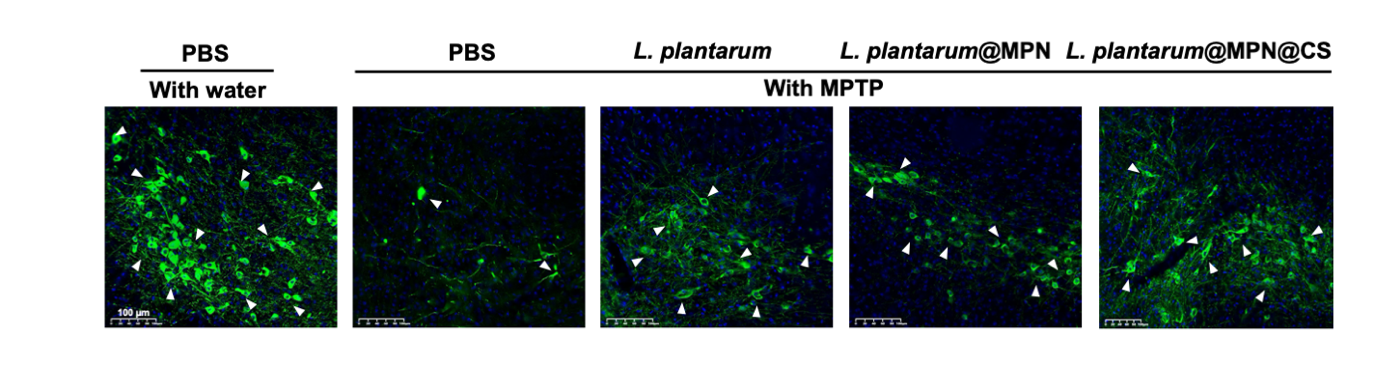


**Figure S16.** **High-magnification images of TH-positive dopaminergic neurons in the substantia nigra.** Green fluorescence represents TH-marked dopaminergic neurons. DAPI was used to stain the nucleus. Scale bar: 100 µm.


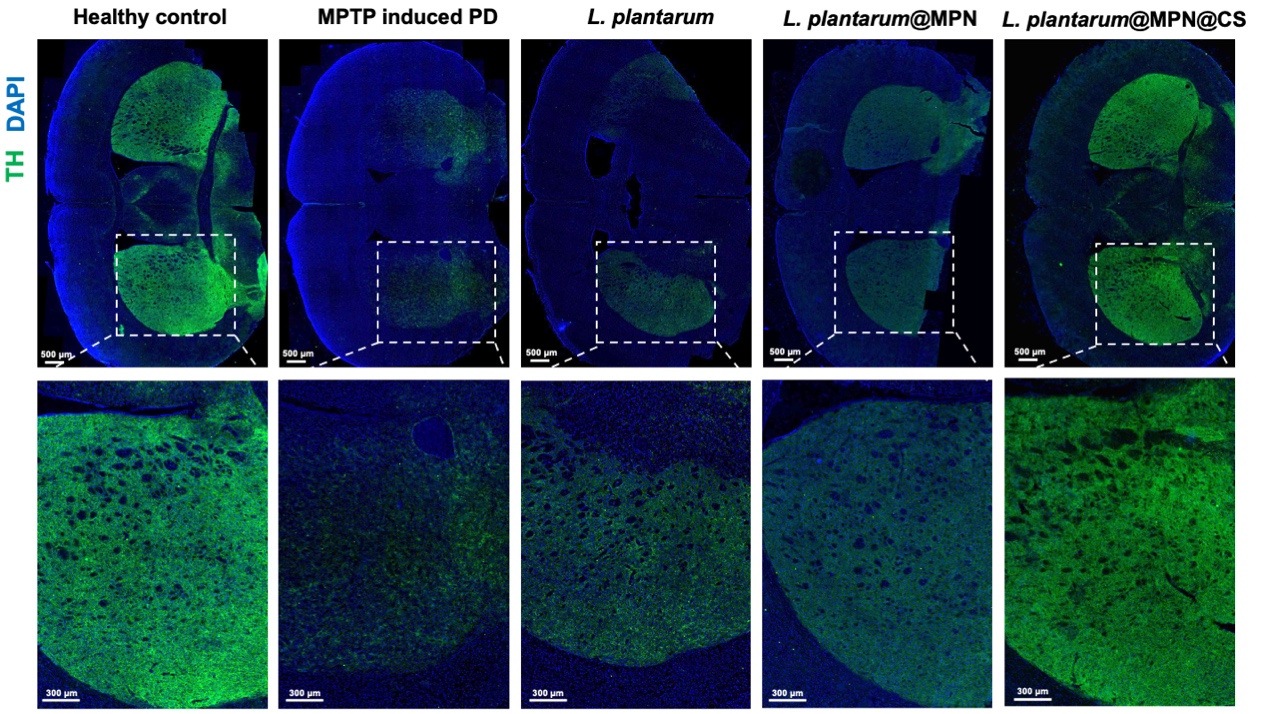


**Figure S17.** ***L. plantarum*@MPN@CS restored the dopaminergic neurons in the striatum.** Representative immunofluorescence images of dopaminergic neuron in the striatum from different groups. Green fluorescence represents TH-marked dopaminergic neurons. DAPI was used to stain the nucleus.


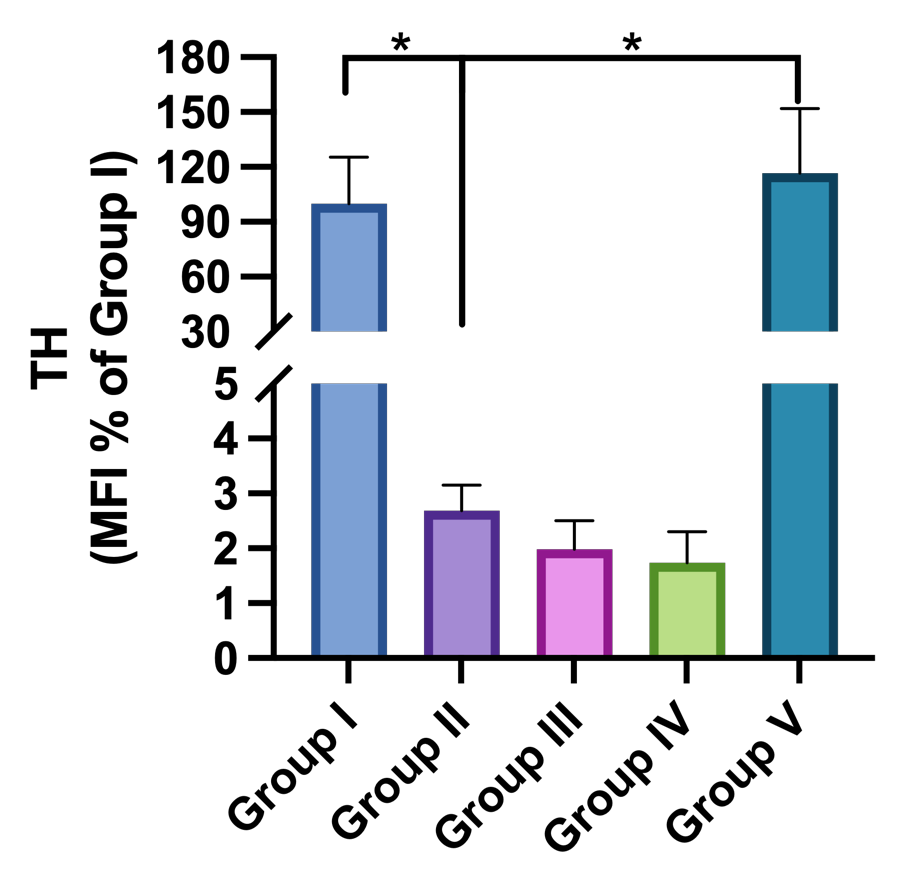


**Figure S18.** **Semiquantitative analysis of the expression of TH in the striatum based on immunofluorescence staining.** Data are presented as means ± SD (n=3 per group). Statistical significance was determined by an unpaired, two-tailed Student’s t-test. *P < 0.05.


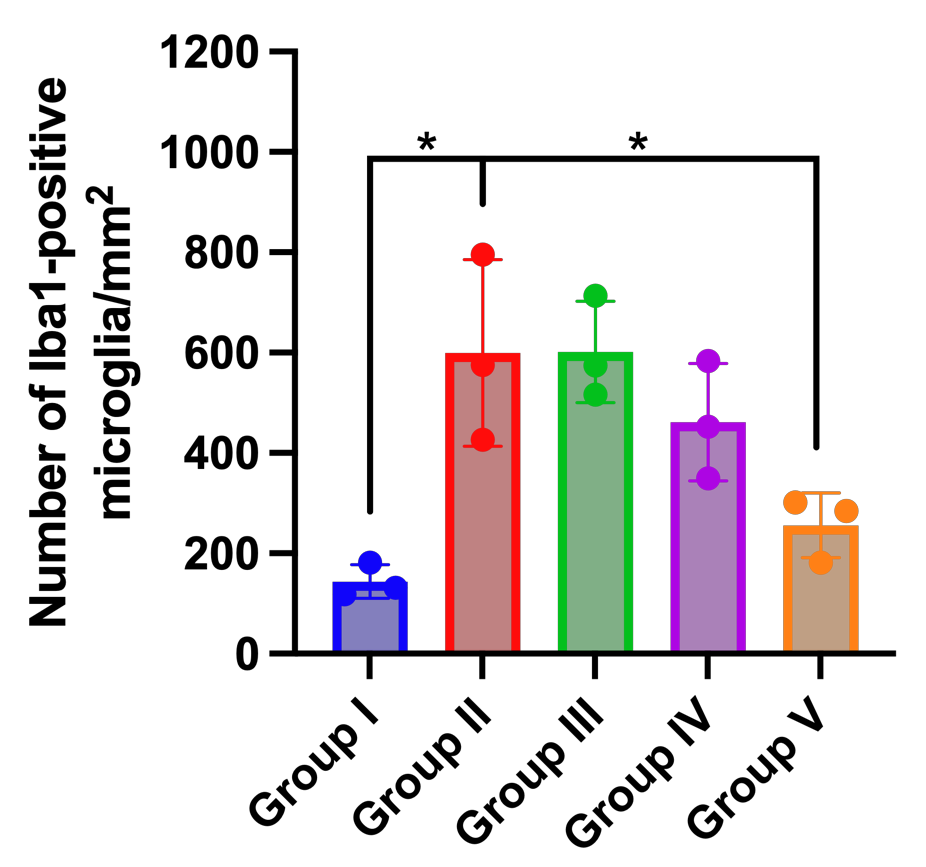


**Figure S19. Quantification of the Iba 1-positive microglia.** Data are presented as means ± SD (n=3 per group). Statistical significance was determined by an unpaired, two-tailed Student’s t-test. *P < 0.05.


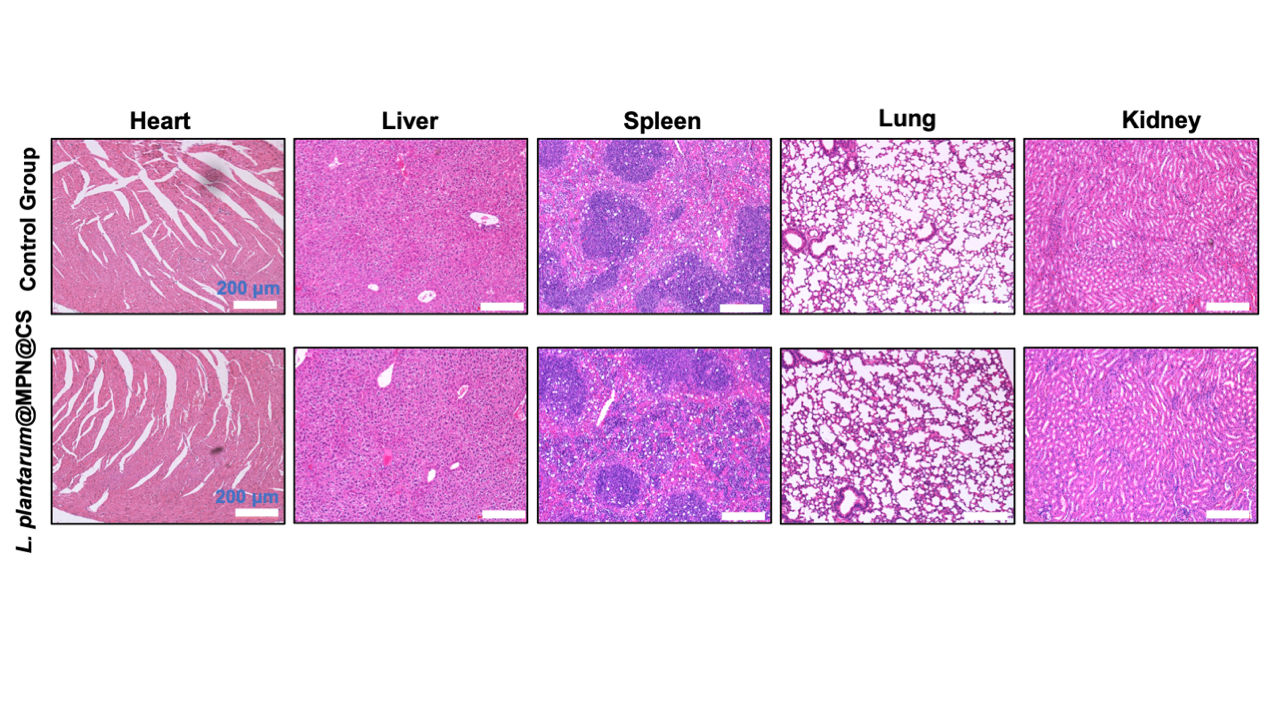


**Figure S20.** **Histopathological evaluation of major organs following prolonged *L. plantarum*@MPN@CS administration.** Tissues were obtained from mice after 30 days of oral administration with 5×10^8 CFU of naked or coated *L. plantarum*. Scale bar: 200 μm.


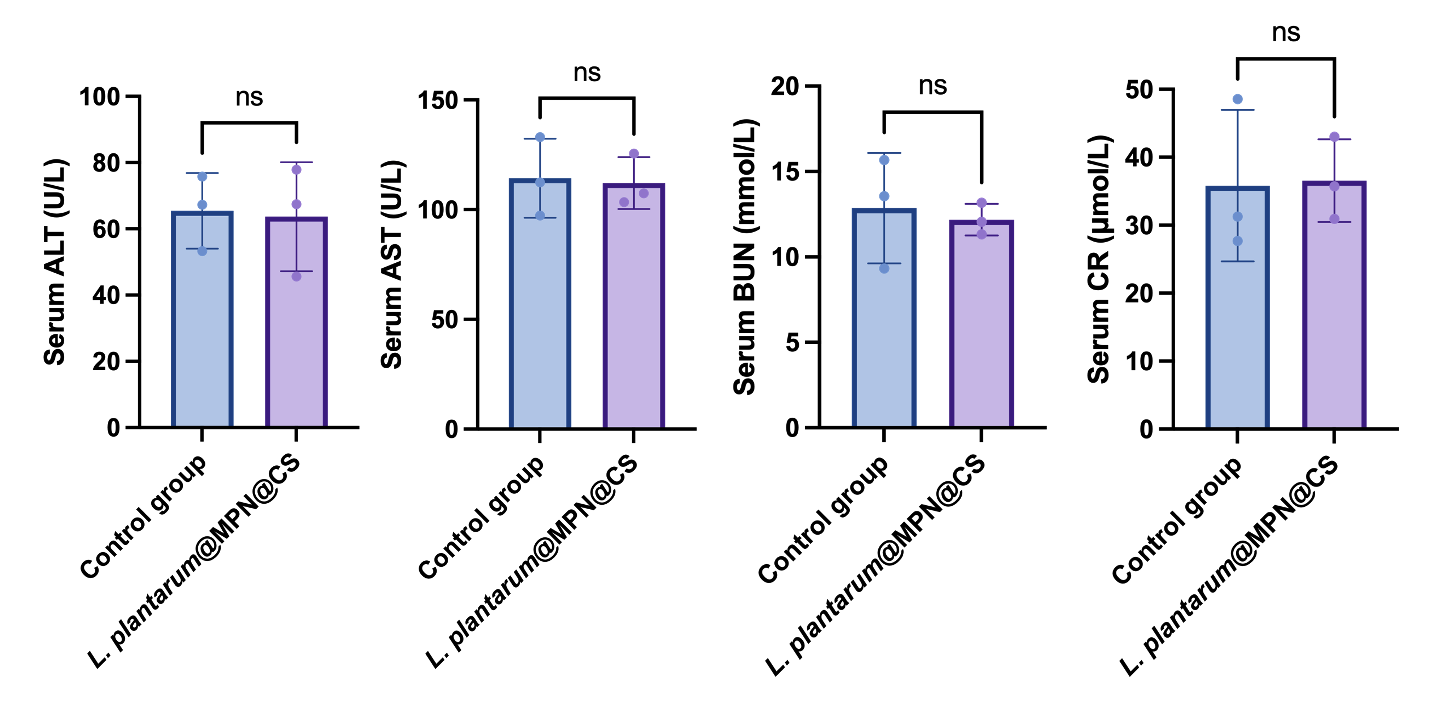
 **Figure S21.** **Biosafety assessment.** Serum levels of ALT, AST, BUN, and CR for mice. Data are presented as means ± SD (n=3 per group). Statistical significance was determined by an unpaired, two-tailed Student’s t-test. ns means non-significant.

**Table S1.** **Histological scoring guideline**

| **Inflammation** | | **Depth of injury** | | **Crypt damage** | | **Percentage involved** | | |
| --- | --- | --- | --- | --- | --- | --- | --- | --- |
| 0 | None | 0 | None | 0 | None | | ×1 | 0~25% |
| 1 | Slight | 1 | Mucosa | 1 | One-third damage | | ×2 | 26%~50% |
| 2 | Moderate | 2 | Mucosa and submucosa | 2 | Two-third damage | | ×3 | 51%~75% |
| 3 | Severe | 3 | Transmural | 3 | Only surface epithelium intact | | ×4 | 76%~100% |
|  |  |  |  | 4 | Entire crypt and epithelium lost | |  |  |

**Table S2.** **Primers used in this study**

| **Primers** | **Forward primer** | **Reversed primer** |
| --- | --- | --- |
| IL-1β | 5'-GCAACTGTTCCTGAACTCAACT-3' | 5'-ATCTTTTGGGGTCCGTCAACT-3' |
| Il-6 | 5'- TAGTCCTTCCTACCCCAATTTCC -3' | 5'-TTGGTCCTTAGCCACTCCTTC-3' |
| TNF-α | 5'- CCCTCACACTCAGATCATCTTCT-3' | 5'- GCTACGACGTGGGCTACAG-3' |
| β-actin | 5'-AGGTGACAGCATTGCTTCTG-3' | 5'-GCTGCCTCAACACCTCAAC-3' |
